# AuCu@CuO_2_ Aerogels with H_2_O_2_/O_2_ Self‐Supplying and Quadruple Enzyme‐Like Activity for *MRSA*‐Infected Diabetic Wound Management

**DOI:** 10.1002/advs.202502391

**Published:** 2025-04-27

**Authors:** Xiaofeng Tan, Nanyun Lin, Sha Yang, Hongyu Gong, Minghui Wang, Na Li, Fen Liu, Dajun Rao, Yingying Wu, Jing Tang, Qinglai Yang

**Affiliations:** ^1^ Department of Anesthesiology Hunan Provincial Maternal and Child Health Care Hospital & NHC Key Laboratory of Birth Defect Research and Prevention & MOE Key Lab of Rare Pediatric Disease Hengyang Medical School University of South China Hengyang Hunan 421001 China; ^2^ Department of Hepatopancreatobiliary Surgery The First Affiliated Hospital & Center for Molecular Imaging Probe Cancer Research Institute Hengyang Medical School University of South China Hengyang Hunan 421001 China; ^3^ Pathology Research Group & Department of Pathology Institute of Basic Disease Sciences & School of Basic Medical Sciences Xiangnan University Chenzhou Hunan 423000 China

**Keywords:** diabetic wound healing, H_2_O_2_ and O_2_ self‐supplying, metallic aerogels, *MRSA* infection, multienzyme‐like activity

## Abstract

Diabetic wound healing presents serious clinical challenges due to the unique wound microenvironment characterized by hyperglycemia, bacterial infection, excessive oxidative stress, and hypoxia. Herein, a copper peroxide (CuO_2_)‐coated AuCu bimetallic aerogel is developed that exhibits quadruple enzyme‐mimicking activity and H_2_O_2_/O_2_ self‐supplying to modulate the complex microenvironment of *methicillin‐resistant staphylococcus aureus (MRSA)*‐infected diabetic wounds. The AuCu@CuO_2_ aerogels demonstrate favorable photothermal properties and mimic four enzyme‐like activities: peroxidase‐like activity for producing toxic reactive oxygen species; catalase‐like activity for decomposing H_2_O_2_ to release O_2_ to relieve oxidative stress and hypoxia; glucose oxidase‐like activity for reducing excessive blood glucose and glutathione peroxidase‐like activity for balancing abnormal glutathione level. The CuO_2_ coating facilitates a continuous and adequate in situ production of H_2_O_2_ within the mildly acidic infection microenvironment, enabling excellent antibacterial activity and reduced blood glucose levels during the initial treatment of infected diabetic wounds. Furthermore, the engineered AuCu@CuO_2_ aerogels not only scavenge elevated ROS during the inflammatory phase but also synergistically generate oxygen to promote wound healing. Overall, the AuCu@CuO_2_ aerogelsmicroenvironment can be activated by the diabetic wound infection microenvironments, alleviating inflammation, reducing hypoxia, lowering blood glucose levels, and enhancing angiogenesis and collagen fiber accumulation, thereby significantly improving diabetic wound healing.

## Graphical abstract

The AuCu@CuO_2_ aerogels demonstrate favorable photothermal properties, four types of enzyme‐like activities, and H_2_O_2_/O_2_ self‐supplying to modulate the complex microenvironment of *MRSA*‐infected diabetic wounds. The results indicate that the AuCu@CuO_2_ aerogel can be activated by the microenvironment of diabetic wound infection, alleviating inflammation, reducing hypoxia, lowering blood glucose levels, and enhancing angiogenesis and collagen fiber accumulation, thereby significantly improving diabetic wound healing.

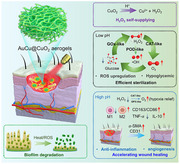

## 1 Introduction

Diabetes, a complex metabolic disease characterized by hyperglycemia, often leads to a range of severe complications, particularly among older individuals and those with obesity.^[^ ^1^, ^2^ ^]^ In diabetic patients, wound infections present significant challenges, as the hyperglycemic microenvironment promotes the production of proinflammatory cytokines, hypoxia, and excessive oxidative stress.^[^ ^3^, ^4^ ^]^ These factors extend the inflammatory phase and delay the proliferation phase of the healing process, resulting in persistent pain, wound maceration, and exudation.^[^ ^5^ ^]^ Furthermore, the prolonged colonization of bacteria, particularly drug‐resistant strains, leads to the formation of resilient 3D biofilms that increase resistance to host immune responses, antibiotics, and external stimuli.^[^ ^6^ ^]^ Notably, elevated blood glucose levels further facilitate bacterial proliferation and biofilm development in diabetic wounds.^[^ ^7^ ^]^ Consequently, there is a significant clinical demand for effective antibiotic‐free therapeutic agents to regulate the infection microenvironment, enabling sterilization and accelerated healing of diabetic wounds.

The wound healing process typically encompasses four consecutive yet overlapping phases: hemostasis, inflammation, proliferation, and tissue remodeling.^[^ ^8^, ^9^ ^]^ During the initial stages, a substantial influx of bacteria into the wound area creates a weakly acidic infection microenvironment (pH = 5.5), characterized by a limited concentration of hydrogen peroxide (H_2_O_2_) and overexpression of glutathione (GSH).^[^ ^10^ ^]^ Damaged blood vessels contribute to the development of chronic hypoxia, which adversely affects the activity of various cytokines and the regeneration of granulation tissue, thereby impeding proliferation and tissue remodeling.^[^ ^11^, ^12^, ^13^ ^]^ Additionally, elevated reactive oxygen species (ROS) levels during the inflammatory phase (pH = 6.5–7.0) can disrupt intercellular signaling pathways, disturb redox homeostasis, and promote the differentiation of M1 macrophages.^[^ ^14^, ^15^ ^]^ Therefore, the dynamic pH levels during the healing of diabetic wounds, largely influenced by glucose concentrations and wound severity, present both challenges and opportunities for effective diabetic wound management.^[^ ^16^, ^17^ ^]^ Consequently, implementing strategies that ensure efficient disinfection in the initial acidic environment, alongside ROS scavenging and hypoxia alleviation in subsequent near‐neutral environments, is rational and effective for eliminating drug‐resistant bacteria and promoting wound healing in diabetic patients.

Various nanozymes have been developed as potential therapeutic agents for the treatment of wound infections. These nanozymes, encompassing pro‐oxidant, anti‐oxidant, and hybrid types, play vital roles in the overlapping phases of diabetic wound healing.^[^ ^18^ ^]^ For instance, nanozymes with peroxidase (POD) and oxidase (OD)‐like activities facilitate the transformation of H_2_O_2_ and O_2_ into highly active ROS.^[^ ^19^, ^20^ ^]^ The generated ROS effectively eradicate bacteria at the wound site by inducing lipid peroxidation in bacterial cell membranes and directly damaging proteins and DNA, thereby circumventing the issue of drug resistance.^[^ ^21^ ^]^ However, ROS‐based therapy confronts challenges due to upregulated GSH levels resulting from anaerobic glycolysis in infected microenvironments.^[^ ^22^, ^23^ ^]^ The GSH peroxidase (GPX)‐like activity of nanozymes has the potential to deplete GSH, thereby enhancing catalytic bactericidal effects.^[^ ^24^, ^25^ ^]^ Additionally, Additionally, glucose oxidase (GOx)‐like activity facilitates the conversion of glucose into gluconic acid and H_2_O_2_, which subsequently generates more ROS to augment antibacterial effects.^[^ ^26^, ^27^ ^]^ Furthermore, nanozymes with catalase (CAT) or superoxide dismutase (SOD)‐like activities demonstrate a unique ability to neutralize ROS in inflammatory microenvironments, thereby mitigating oxidative stress and hypoxia to promote wound healing.^[^ ^28^, ^29^, ^30^, ^31^ ^]^ An ample supply of O_2_ can enhance angiogenesis, collagen deposition, and cell proliferation, accelerating wound repair.^[^ ^32^ ^]^ Therefore, the rational design of multifunctional nanozymes exhibiting both pro‐oxidant and anti‐oxidant properties, tailored to the distinct physiological characteristics present at various stages of infected diabetic wound healing, could optimize sterilization efficacy and facilitate wound healing.

Metallic aerogel‐based nanozymes, characterized by abundant metallic active sites and hierarchical pore structures, provide robust activity and stability for ROS‐related sterilization.^[^ ^33^ ^]^ Nevertheless, the antibacterial efficacy of current treatments is constrained by insufficient levels of H_2_O_2_, overexpressed GSH, and severe hypoxia in the infection region. Although exogenous H_2_O_2_ is commonly utilized to augment continuous ROS generation during sterilization, its application can damage healthy tissues.^[^ ^34^, ^35^ ^]^ Consequently, it is imperative to design multienzyme‐like nanozymes capable of enhancing antimicrobial activity without relying on exogenous H_2_O_2_. Copper peroxide (CuO_2_), which demonstrates efficient self‐supplying H_2_O_2_ capabilities under acidic conditions, has been proposed to enhance ROS‐mediated sterilization.^[^ ^36^ ^]^ Moreover, the generated H_2_O_2_ can be converted into O_2_ through catalase‐like activity, thereby alleviating hypoxia in the wound‐healing process.

**Scheme 1:**
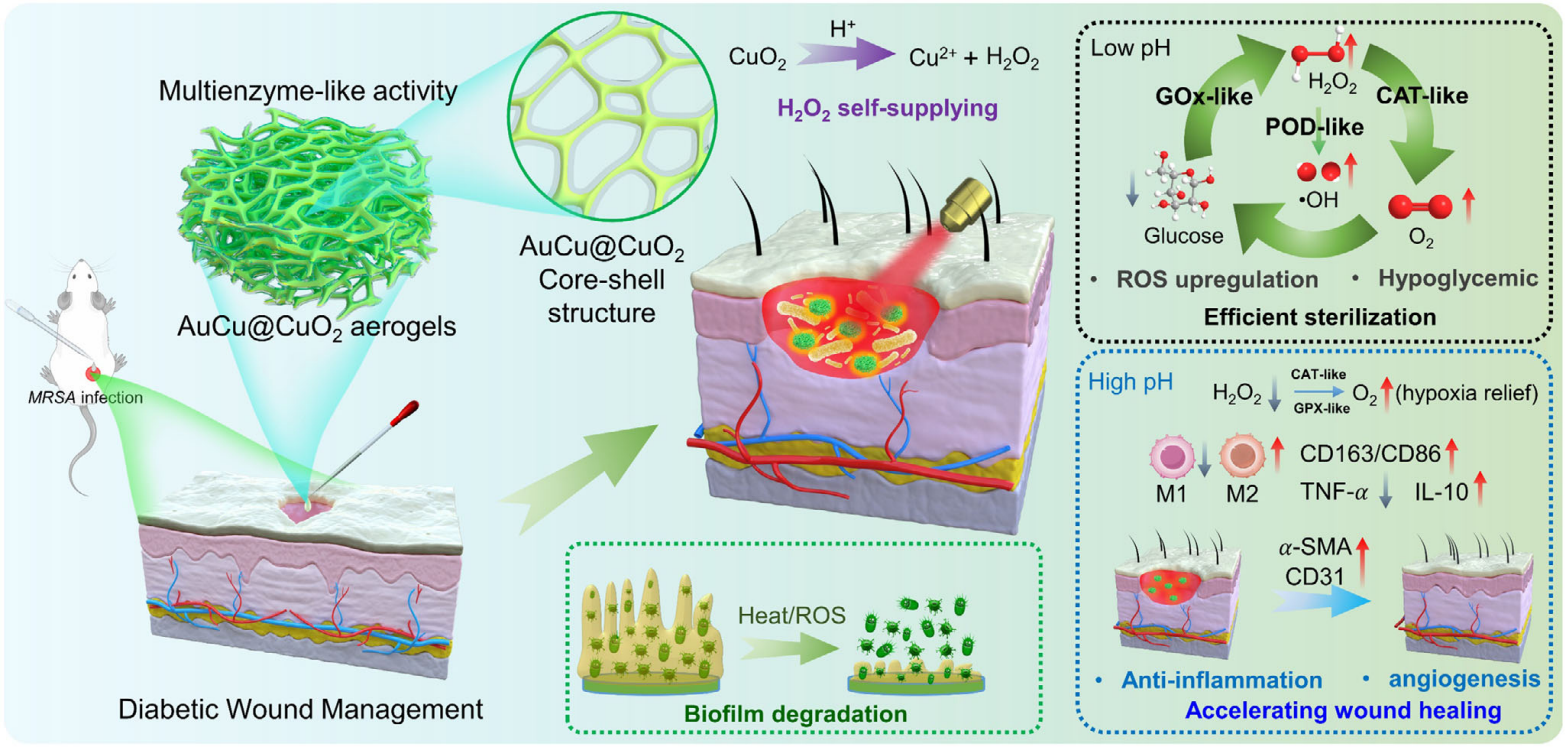
Schematic illustration of treatment mechanism of AuCu@CuO_2_ aerogels with H_2_O_2_/O_2_ self‐supply, glucose depletion, and ROS upregulation for efficient sterilization and healing acceleration in diabetic wound management.

Herein, a CuO_2_‐coated AuCu aerogel nanozyme with H_2_O_2_/O_2_ self‐supplying and quadruple enzyme‐mimicking activity was reported for the management of *MRSA*‐infected diabetic wounds. The AuCu@CuO_2_ aerogels demonstrate satisfactory photothermal conversion efficiency (*η* = 63.9%) and photostability. Moreover, the AuCu@CuO_2_ aerogels exhibit multiple enzyme‐like activities, including POD‐like, GPX‐like, CAT‐like, and GOx‐like activity, effectively regulating the microenvironment of diabetic wound infections and the healing process. The CuO_2_ coating facilitates a constant and sufficient in situ supply of H_2_O_2_ for the reinforcement of POD‐like activity to produce toxic ROS under slightly acidic infection environments. The GOx‐like activity enables the reduction of glucose levels, resulting in the production of H_2_O_2_ and gluconic acid, thereby enhancing the antibacterial effect by inhibiting bacterial growth and biofilm formation. Moreover, the excess GSH present in the infection site can be depleted by the GPX‐like activity of AuCu@CuO_2_ aerogels to enhance the antibacterial and healing effect by reducing the protective mechanisms of bacteria against oxidative stress and improving the efficacy of the immune response. Furthermore, the generated H_2_O_2_ could be decomposed into O_2_ by the CAT‐like activity of AuCu@CuO_2_ aerogels, thereby mitigating oxidative stress and hypoxia to enhance cellular respiration, angiogenesis, and immune function. The multifunctional activity of AuCu@CuO_2_ aerogels constructs a unique cascaded catalytic system to achieve synergistic sterilization, which is less prone to inducing bacterial resistance.^[^ ^37^, ^38^, ^39^, ^40^ ^]^ Both in vivo and in vitro experiments indicate that AuCu@CuO_2_ aerogels offer a gentle yet effective approach for curbing bacterial growth and mitigating antibiotic resistance. Furthermore, the designed AuCu@CuO_2_ aerogels facilitate skin regeneration by enhancing epithelialization, collagen deposition, and angiogenesis, as evidenced by the secretion of relevant cytokines(**Scheme** **1**).

## 2 Results and Discussion

### 2.1 Preparation and Characterization of AuCu@CuO_2_ Aerogels

**Figure 1:**
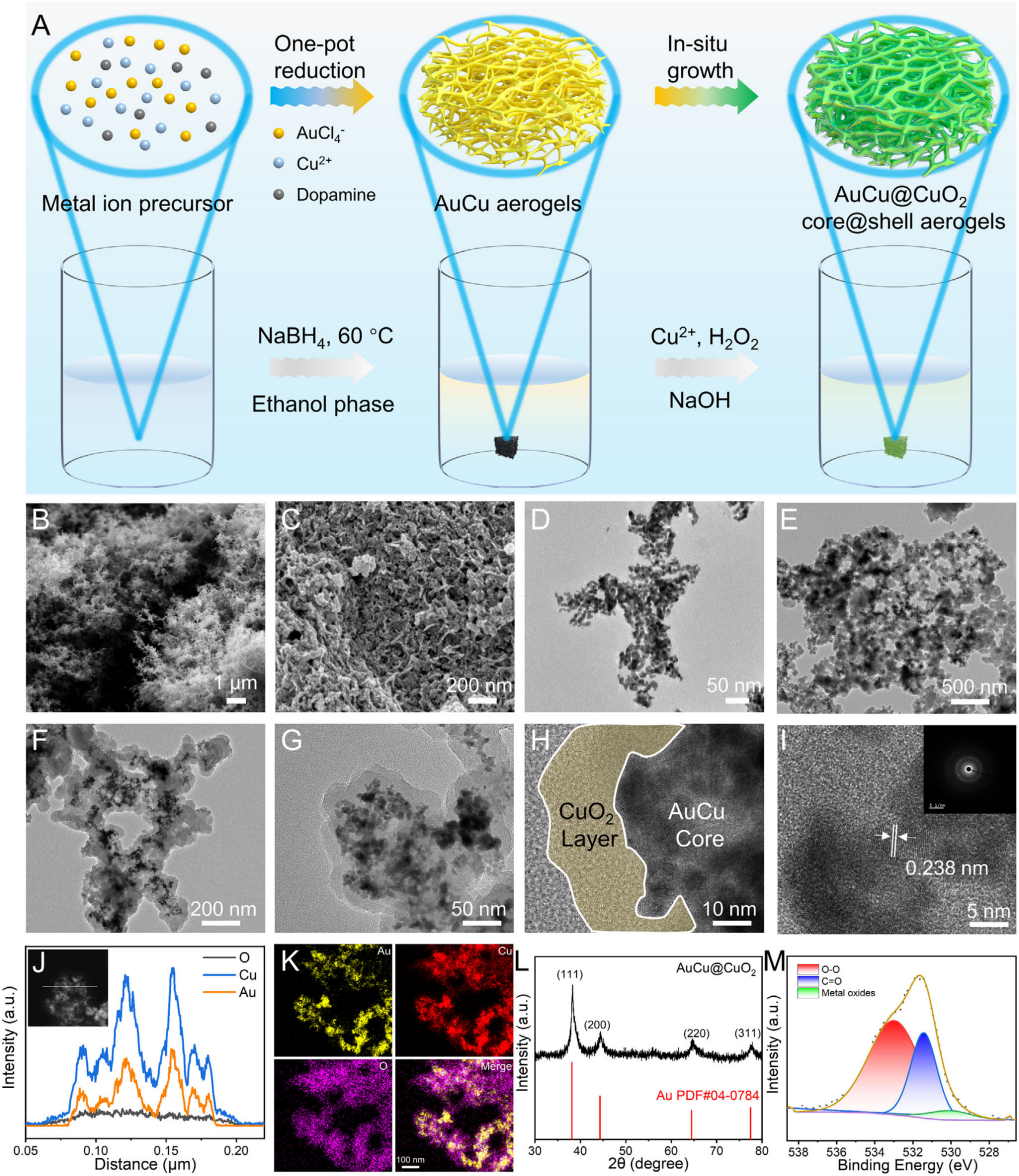
A) The preparation process of AuCu@CuO_2_ aerogels. B,C) SEM images and D–G) TEM images with different magnifications of AuCu and AuCu@CuO_2_ aerogels. H–I) HRTEM images, SAED pattern, J) line‐scan, K) element mapping of Au, Cu, O, and their merge in AuCu@CuO_2_ aerogels. L) XRD pattern and M) XPS spectra of O 1s for AuCu@CuO_2_ aerogels.

The AuCu aerogels were initially synthesized using NaBH_4_ reduction in the ethanol. Subsequently, the CuO_2_ was coated on the AuCu aerogels through an in‐situ growth method (**Figure** **1**A). After thorough washing and freeze‐drying, the morphological characteristics of the AuCu@CuO_2_ aerogels were analyzed using scanning electron microscopy (SEM) and transmission electron microscopy (TEM). As shown in Figure 1B,C, the SEM images reveal that both AuCu and AuCu@CuO_2_ aerogels exhibit a spongy structure with a 3D channel and pore, providing a high specific surface area for substrate transfer. The TEM images of AuCu aerogels demonstrate interconnected metallic nanowire structures (Figure 1D). In contrast, the TEM images of AuCu@CuO_2_ aerogels display that the CuO_2_ layer envelops the exterior of the AuCu aerogel structure (Figure 1E–H). High‐resolution TEM images indicate a clear demarcation of the CuO_2_ layer, with an estimated thickness of approximately 15 nm, and reveal the distributed lattice spacing of 0.238 nm along with the AuCu metallic nanowire. Additionally, the selected area electron diffraction (SAED) pattern exhibits distinct concentric diffraction loops, demonstrating the polycrystalline structure of the AuCu@CuO_2_ aerogels (Figure 1I). The composition of AuCu@CuO_2_ aerogels was further examined by high‐angle annular dark‐field scanning transmission electron microscopy (HAADF‐STEM). The compositional line‐scanning profiles show an absence of Cu and O signals in the outer region, while the Au signal is present in the inner region, thereby confirming the core‐shell structure (Figure 1J). The corresponding elemental mappings illustrate a homogeneous distribution of Au, Cu, and O elements within the AuCu@CuO_2_ aerogels, indicating successful encapsulation of AuCu by CuO_2_ (Figure 1K).

The crystal structure and composition of AuCu@CuO_2_ aerogels were analyzed using X‐ray diffraction (XRD). The results revealed four distinct diffraction peaks at 38.25°, 44.46°, 64.69°, and 77.71°, which correspond to the standard peaks of Au (PDF#04‐0784) (Figure 1L). High‐resolution X‐ray photoelectron spectroscopy (XPS) was employed to elucidate the elemental valence states of AuCu@CuO_2_ aerogels. The high‐resolution XPS spectra of O 1s displayed three peaks at 529.9, 531.4, and 533.0 eV, attributed to Cu─O, C═O, and O─O bonds, respectively, indicating the presence of polyvinylpyrrolidone and copper peroxide. For the Au element, the peaks observed at 87.3 and 83.6 eV correspond to Au 4f5/2 and Au 4f7/2, respectively, suggesting that the valence state of Au is predominantly metallic. Additionally, the valence state of Cu was found to be multivalent, as indicated by the Cu 2p spectra. This spectrum exhibited two principal peaks at 933.5 and 953.5 eV, along with satellite peaks at 941.3, 943.7, and 962.1 eV, respectively (Figure S1, Supporting Information).

### 2.2 Multienzyme‐Like Activity and Photothermal Properties of AuCu@CuO_2_ Aerogels

The AuCu@CuO_2_ aerogels can generate H_2_O_2_ and Cu^2+^ under the weakly acidic condition. The Cu^2+^ released from the CuO_2_ was first quantified using a copper detection kit. As shown in Figure S2 (Supporting Information), the released Cu^2+^ concentration was determined using a standard working concentration curve of the detection kit. The released Cu^2+^ from the AuCu@CuO_2_ aerogels was estimated to be approximately 3.1 mg L^−1^. The KMnO_4_ colorimetric assay was employed to verify the release of H_2_O_2_ from AuCu@CuO_2_ aerogels because the released H_2_O_2_ can react with the pink MnO_4_^−^ to form colorless Mn^2+^. As illustrated in Figure S3 (Supporting Information), the reaction solution becomes transparent, and the characteristic peaks vanish upon the addition of AuCu@CuO_2_ aerogels or CuO_2_, indicating their ability to release H_2_O_2_ in an acidic environment.

**Figure 2:**
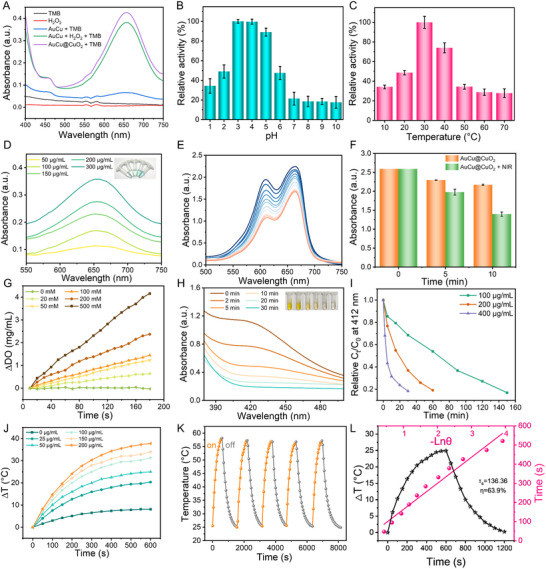
A) UV−vis absorption spectra for evaluating the POD‐like activity using the TMB reaction system. The effect of B) pH, C) temperature, and D) AuCu@CuO_2_ aerogels concentrations on POD‐like activity. E) MB degradation spectra over time using AuCu@CuO_2_ aerogels. F) laser‐enhanced POD‐like activity in different times based on the MB degradation experiments. G) O_2_ generation under different concentrations of H_2_O_2_ based on AuCu aerogels. H) The absorption spectra of GSH consumption by AuCu@CuO_2_ aerogels and I) GSH consumption rate under different concentrations of AuCu@CuO_2_ aerogels. J) The temperature‐raising curves at different concentrations of AuCu@CuO_2_ aerogels. K) The temperature changes during the 5 on/off laser cycles. L) The heating curves and linear cooling time and fitting of time versus the negative natural logarithm of driving force temperature. (The error bar represents the standard deviation from the repeated experiments after three times).

The POD‐like activity of AuCu@CuO_2_ aerogels was assessed using the TMB colorimetric assay. As shown in **Figure** **2**A, the AuCu@CuO_2_ aerogels + TMB group exhibited a maximum characteristic peak at 652 nm, while the AuCu aerogels + TMB group displayed a weak signal intensity at 652 nm. The analysis for the effect of reaction pH and temperature on POD‐like activity showed that AuCu@CuO_2_ aerogels have the strongest activity at pH 4 and 30 °C, aligning with the acid‐responsive performance of CuO_2_ (Figure 2B,C). In addition, as the concentration of AuCu@CuO_2_ aerogels increased, the POD‐like activity increased, as indicated by the enhanced absorbance at 652 nm (Figure 2D). Furthermore, methyl blue (MB) degradation experiments were conducted to validate the generation of hydroxyl radicals (·OH). As demonstrated in Figure 2E, the gradual decrease in absorbance at 650 nm is attributed to the generation of ·OH by the AuCu@CuO_2_ aerogels. Notably, the reduction in absorbance at 650 nm became more pronounced after laser irradiation, suggesting that laser irradiation enhanced the production of ·OH by the AuCu@CuO_2_ aerogels (Figure 2F).

Excess H_2_O_2_ can oxidize intracellular biomolecules, leading to cellular damage, while the hypoxic environment at the wound site hinders healing. CAT mimics can decompose H_2_O_2_ into harmless water and O_2_, preventing the accumulation of H_2_O_2_ and reducing oxidative damage to cells while also delivering O_2_ to the wound site to enhance cellular activity and collagen synthesis, thereby promoting tissue regeneration. The CAT‐like activity of AuCu and AuCu@CuO_2_ aerogels was demonstrated by detecting H_2_O_2_‐triggered O_2_ production using a dissolved oxygen meter. As shown in Figure S4 (Supporting Information), the AuCu and AuCu@CuO_2_ aerogels exhibited similar CAT‐like activity, and the generated H_2_O_2_ was progressively decomposed over time to yield O_2_, as indicated by the gradual formation of bubbles. Moreover, the O_2_ production rate increased with increasing H_2_O_2_ concentration (Figure 2G).

GSH overexpression is detrimental to the POD‐like activity of nanozyme. Therefore, depleting GSH through GPX‐like activity at the infection site could enhance the antibacterial effect of nanozymes. The DTNB probe was utilized to assess the GPX‐like activity of AuCu@CuO_2_ aerogels. Briefly, when only DTNB and GSH are present, a yellow compound, 5‐thio‐2‐nitrobenzoic acid, is formed, exhibiting a characteristic peak at 412 nm. After incubating AuCu@CuO_2_ aerogels with GSH over time, the residual GSH was detected quantitatively by DTNB. As shown in Figure 2H and Figure S5 (Supporting Information), the characteristic peaks corresponding to 5‐thio‐2‐nitrobenzoic acid gradually decreased with increasing reaction time, demonstrating the efficient GPX‐like activity of AuCu@CuO_2_. Notably, the consumption of GSH increased with the higher concentrations of AuCu@CuO_2_ aerogels (Figure 2I). The conversion rate of GSH reached 82% after incubating with AuCu@CuO₂ aerogels at 200 µg mL^−1^.

The high concentration of glucose in diabetics can lead to excessive glycation and chronic wound inflammation. Interestingly, glucose oxidase (GOx) can convert glucose into gluconic acid and H_2_O_2_, alleviating high glucose levels and lowering the wound pH. The GOx‐like activity of AuCu@CuO_2_ aerogels was verified using HRP and TMB to detect the produced H_2_O_2_. As revealed in Figure S6 (Supporting Information), the reaction solution after incubation of AuCu@CuO_2_ aerogels and glucose showed a distinct absorption peak at 652 nm, with the solution turning blue. However, AuCu@CuO_2_ aerogels and glucose alone did not show the characteristic peak after incubation, indicating their efficient GOx‐like activity. Moreover, this activity is concentration‐dependent, gradually enhancing with increasing concentrations of AuCu@CuO_2_ aerogels. (Figure S7, Supporting Information).

As shown in Figure S8 (Supporting Information), AuCu@CuO_2_ aerogels exhibited broad absorption, demonstrating excellent NIR‐II photothermal conversion potential. Thermal imaging was used to collect temperature images and change curves of different concentrations of AuCu@CuO_2_ aerogels after laser irradiation (1064 nm, 10 min, 1 W cm^−2^) (Figure 2J and Figure S9, Supporting Information). The results indicated that the temperature of AuCu@CuO_2_ aerogels (100 µg mL^−1^) increased by 31.3 °C within 10 min, with the temperature change rising with increasing concentrations of the AuCu@CuO_2_ aerogels. Additionally, the temperature increases of AuCu@CuO_2_ aerogels were enhanced by higher power densities (Figure S10, Supporting Information). Moreover, no significant fluctuations were observed during the five cycles of laser on/off switching tests, indicating that the AuCu@CuO_2_ aerogels possess significant photothermal stability (Figure 2K). Finally, the photothermal conversion efficiency (*η*) of AuCu@CuO_2_ aerogels was calculated to be 63.9% (Figure 2L), which is superior to that of the most reported photothermal agents^[^ ^41^, ^42^ ^]^ (Table S1, Supporting Information), indicating an excellent photothermal sterilization potential for AuCu@CuO_2_ aerogels.

### 2.3 In Vitro Antibacterial Performances of AuCu@CuO_2_ Aerogels

**Figure 3:**
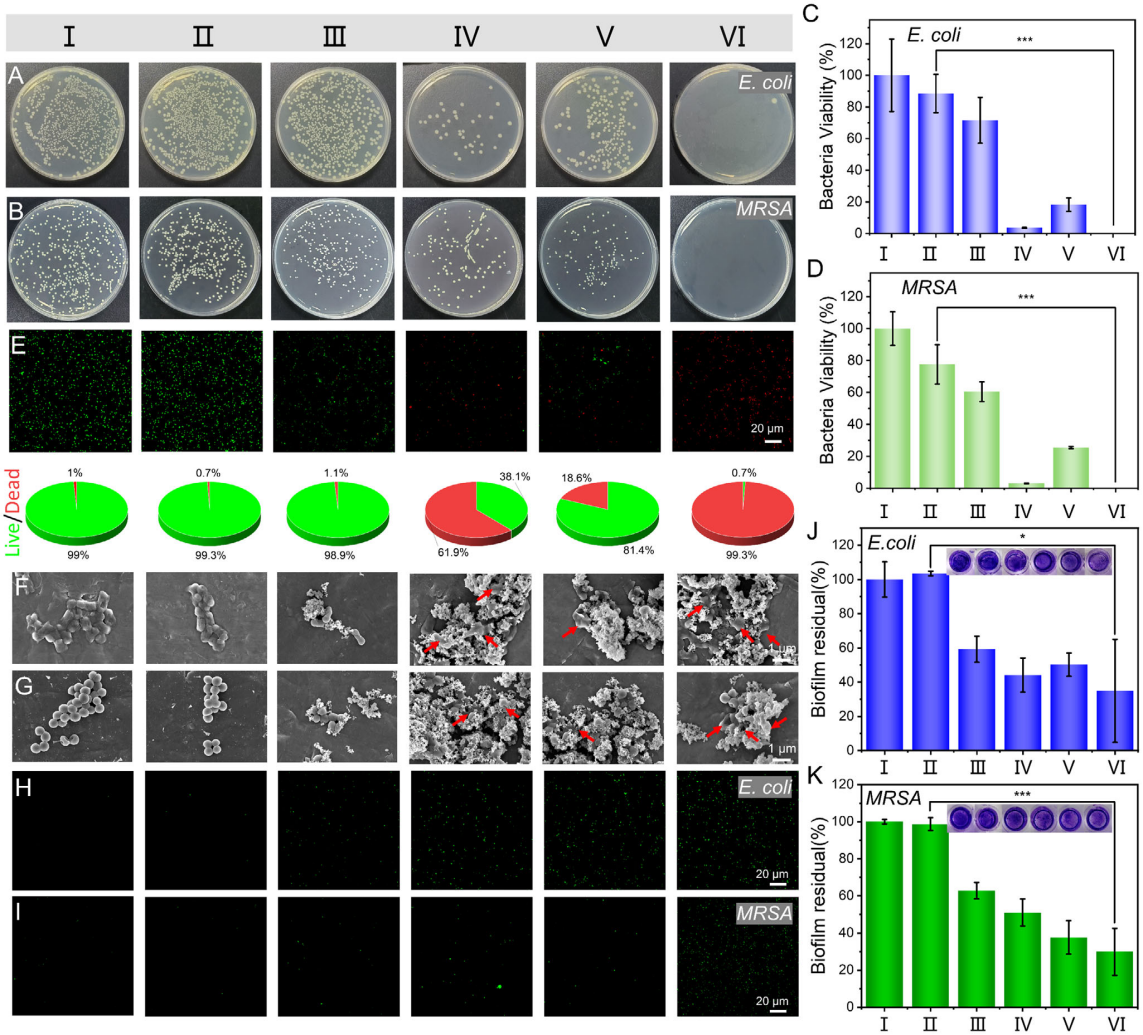
Photographs and bacteria viability of A,C) *E. coli* and B,D) *MRSA* colonies after different treatments. E) Live/dead staining images and percentage of *MRSA after different treatments*. SEM images and ROS fluorescence staining of F,H) *E. coli* and G,I) *MRSA after different treatments*. The percentages of biofilms for J) *E. coli* and K) *MRSA* following different treatments, with visual representations of biofilms stained with crystal violet. (AuCu@CuO_2_ aerogels: 100 µg mL^−1^; NIR: 1064 nm, 1W cm^−2^, 10 min). I) PBS, II) PBS + NIR, III) AuCu aerogels, IV) AuCu aerogels + NIR, V) AuCu@CuO_2_ aerogels, VI) AuCu@CuO_2_ aerogels + NIR. (The error bar represents the standard deviation from the repeated experiments after three times. *P*‐value: *P < 0.05, **P < 0.01, ***P < 0.001).

*E. coli* and *MRSA* were selected as model bacteria representing Gram‐negative and Gram‐positive strains to evaluate the in vitro full‐spectrum antibacterial performances of AuCu@CuO_2_ aerogels. First, the viability of bacterial colonies was investigated through plate counting experiments after different treatments: I) PBS, II) PBS + NIR, III) AuCu aerogels, IV) AuCu aerogels + NIR, V) AuCu@CuO_2_ aerogels, VI) AuCu@CuO_2_ aerogels + NIR. As shown in **Figure** **3**A–D, a large number of bacterial colonies were observed in the PBS group and the AuCu aerogels, indicating that neither irradiation nor AuCu aerogels alone can effectively restrict bacterial growth. In the AuCu aerogels + NIR group and the AuCu@CuO_2_ aerogels group, bacterial colony formation was inhibited, while a small number of residual colonies were still observed. The AuCu@CuO_2_ aerogels + NIR group indicated an inhibition rate against bacteria of over 99%, demonstrating the synergistic photothermal and nanozymes antimicrobial effect of AuCu@CuO_2_ aerogels.

To further demonstrate the synergistic antimicrobial properties of the AuCu@CuO_2_ aerogels, live/dead bacterial staining was performed with different treatments using a live/dead bacterial staining kit (fluorescent dye N01 for labeling live bacteria and PI for labeling dead bacteria). The results showed that many dead bacteria appeared as red fluorescence in the AuCu@CuO_2_ aerogels + NIR group, consistent with the bacterial colony counting experiments (Figure 3E). Furthermore, the morphology and corresponding ROS concentrations of bacteria under different treatments were examined. SEM was used to observe the morphology of *E. coli* and *MRSA*. The findings revealed that after treatment with PBS or AuCu aerogels, the bacteria exhibited clear surfaces and intact cell walls. In the AuCu@CuO_2_ aerogels group, the cell wall surfaces of both bacteria showed slight collapse, while the major structure of most bacterial cells remained intact, indicating that the multienzyme‐like activity alone was insufficient for complete sterilization. In contrast, the AuCu aerogels + NIR treatment caused the skeletal structures of the bacteria to become significantly wrinkled, while the AuCu@CuO_2_ aerogels + NIR treatment led to complete disruption of the bacteria, with leakage of intracellular cytoplasmic components (Figure 3F,G).

Additionally, the DCFH‐DA probe was used to confirm the changes in ROS levels within bacteria under different treatment conditions. The results showed that the fluorescence signal in the AuCu@CuO_2_ aerogels + NIR groups were the strongest (Figure 3H,I), indicating that the combined photothermal and nanozyme properties of AuCu@CuO_2_ aerogels can produce substantial quantities of ROS. To investigate the biofilm degradation ability of AuCu@CuO_2_ aerogels, crystal violet staining experiments were conducted under different treatments. The biofilm degradation rate for the AuCu aerogels + NIR and AuCu@CuO_2_ aerogels groups was determined to be 50–60%. However, the biofilm degradation rate for the AuCu@CuO_2_ aerogels + NIR reached over 70% (Figure 3J,K). Additionally, the 3D confocal laser scanning microscopy (CLSM) results visualized the effective biofilm‐disrupting effect of AuCu@CuO_2_ aerogels. Compared with the PBS group, a large amount of red fluorescence and thinning biofilms were observed in the AuCu@CuO_2_ aerogels + NIR group, indicating the destructive effect on *MRSA* biofilms effectively. (Figures S11 and S12, Supporting Information).

### 2.4 In Vivo Antibacterial Capability of AuCu@CuO_2_ Aerogels

**Figure 4:**
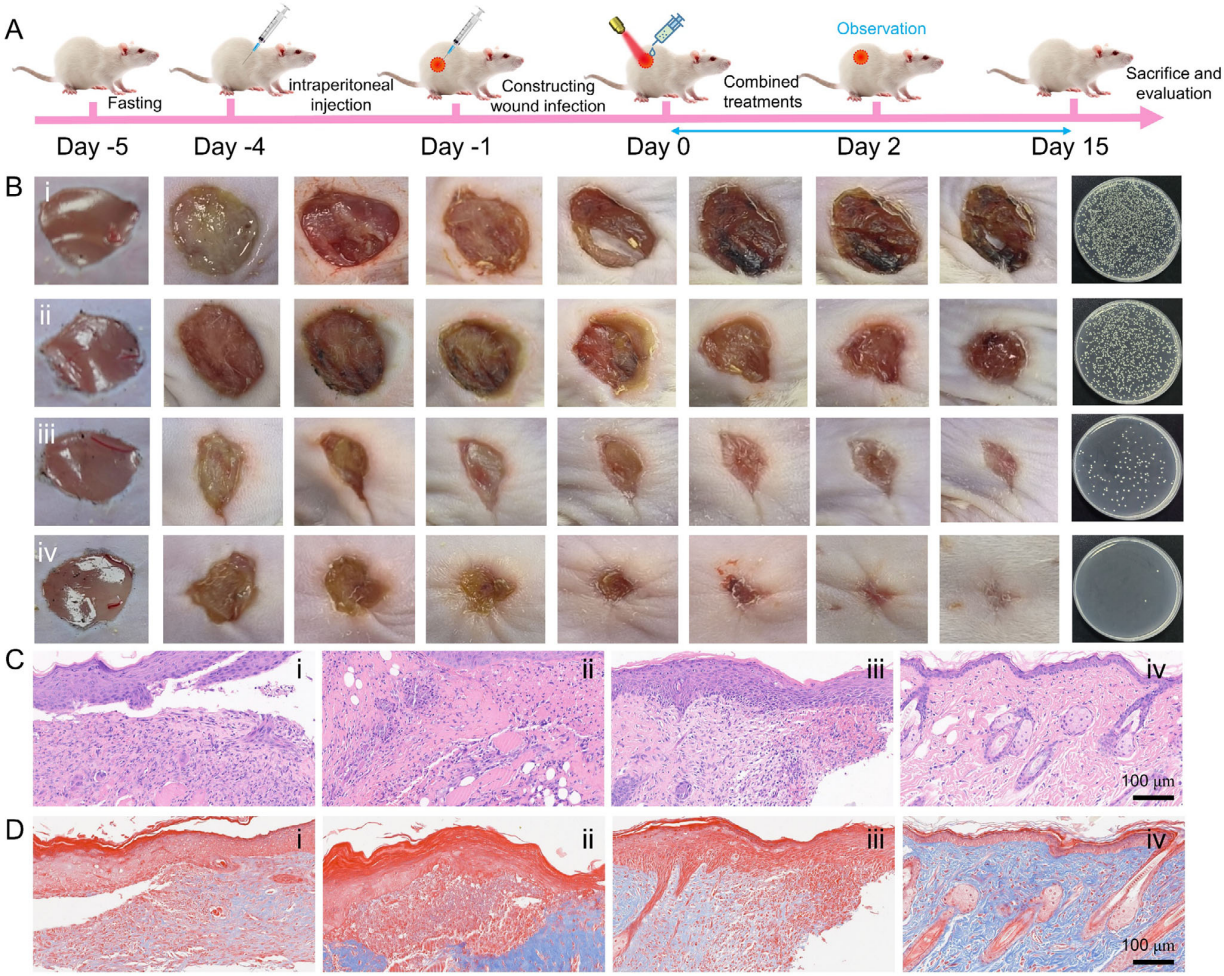
A) The construction diagram of the diabetic wound model infected with *MRSA*. B) Wound size development during 14 d and bacterial colonies cultured in wound skin tissue of *MRSA*‐infected executed mice after different treatments. C) H&E and D) Masson staining of skin tissues with different treatments, scale bar: 100 µm. i) PBS, ii) PBS + NIR, iii) AuCu@CuO_2_ aerogels, iv) AuCu@CuO_2_ aerogels + NIR.

Combining the excellent antibacterial activity of AuCu@CuO_2_ aerogels in vitro, we established a cutaneous injury model in diabetic mice infected with *MRSA* to simulate a chronic wound environment and evaluate the in vivo antibacterial efficacy of AuCu@CuO_2_ aerogels. Using streptozotocin (STZ) chemical induction in KM mice to induce diabetes, then an 8 mm diameter wound was created on the dorsal skin, followed by the inoculation of an *MRSA* suspension into the wound. After 24 hours, to ensure successful wound infection, the KM mice were segregated into four distinct therapeutic groups: (i) PBS, (ii) PBS + NIR, (iii) AuCu@CuO_2_ aerogels, and (iv) AuCu@CuO_2_ aerogels + NIR (**Figure** **4**A). Temperature changes in the wounds were recorded using an infrared thermographic camera (Figure S13, Supporting Information). The temperature at the wound site in the AuCu@CuO_2_ aerogels + NIR group rapidly increased during laser irradiation, indicating their excellent in vivo photothermal performance. The changes in wound size over 13 days showed that the wound of the AuCu@CuO_2_ aerogels + NIR group healed better than those of the AuCu@CuO_2_ aerogels and PBS groups, suggesting that combined therapy can significantly mitigate the risk of wound infections and enhance the wound healing process (Figure 4B and Figure S14, Supporting Information).

After 13 d, the mice were humanely sacrificed to obtain wound tissue for further analysis. The bacterial residue at the wound site was assessed using the plate counting method. The results demonstrated that the bacterial colony in the AuCu@CuO_2_ aerogels + NIR group was markedly diminished compared to the other groups, exhibiting the excellent antibacterial capability of AuCu@CuO2 aerogels. In vivo histological evaluation of wound tissues was performed using H&E and Masson staining. Compared to the PBS group, the AuCu@CuO_2_ aerogels + NIR group exhibited more epidermal restoration, a greater number of hair follicles, and denser collagen fibers (Figure 4C,D).

**Figure 5:**
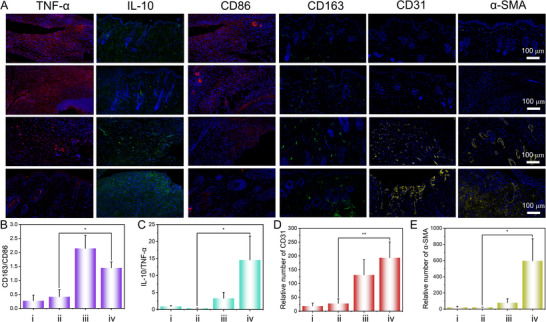
A) Representative immunofluorescence of tissues and quantitative analysis of B) CD163/CD86, C) TNF‐𝛼/IL‐10, D) CD31, (E) 𝛼‐SMA in different groups. i) PBS, ii) PBS + NIR, iii) AuCu@CuO_2_ aerogels, and iv) AuCu@CuO_2_ aerogels + NIR. (The error bar represents the standard deviation from the repeated experiments after three times. *P*‐value: *P < 0.05, **P < 0.01, ***P < 0.001).

Furthermore, immunohistochemical staining was employed to evaluate further the healing process of skin tissue in diabetic wounds treated with AuCu@CuO_2_ aerogels (**Figure** **5**A). The diabetic wound environment tends to elicit higher expression of pro‐inflammatory cytokines. To confirm the impact of AuCu@CuO_2_ aerogels on in vivo inflammation regulation, two phenotypic markers, CD86 (M1 macrophages) and CD163 (M2 macrophages), were evaluated. The results indicated that the CD163/CD86 ratio in the AuCu@CuO_2_ aerogels + NIR group was significantly higher than in the PBS group (Figure 5B), suggesting polarization of macrophages from pro‐inflammatory M1 to pro‐healing M2 types. The evaluation of pro‐inflammatory (TNF‐α) and anti‐inflammatory (IL‐10) cytokine expression after treatments was further conducted. A significant decrease in pro‐inflammatory factors (TNF‐α) and an increase in anti‐inflammatory factors (IL‐10) were observed in the AuCu@CuO_2_ aerogels + NIR groups compared to other groups (Figure 5C), which suggests that AuCu@CuO_2_ aerogels under irradiation are more effective in reducing inflammation of wounds. Angiogenesis is important for the reconstruction of infected diabetic tissue. The CD31 and α‐smooth muscle actin (α‐SMA) factors were typical markers to assess blood vessel formation. The results indicated that CD31 and α‐SMA expression was higher following treatment with AuCu@CuO_2_ aerogels + NIR compared to other groups, indicating improved vascular density and a high pro‐vascularization capability to support wound healing (Figure 5D,E).

Overall, these results demonstrate that combined therapy with AuCu@CuO_2_ aerogels achieves efficient antibacterial effects, downregulation of the inflammatory response, and pro‐vascularization capability, thereby facilitating the healing of chronic diabetic wounds.

**Figure 6:**
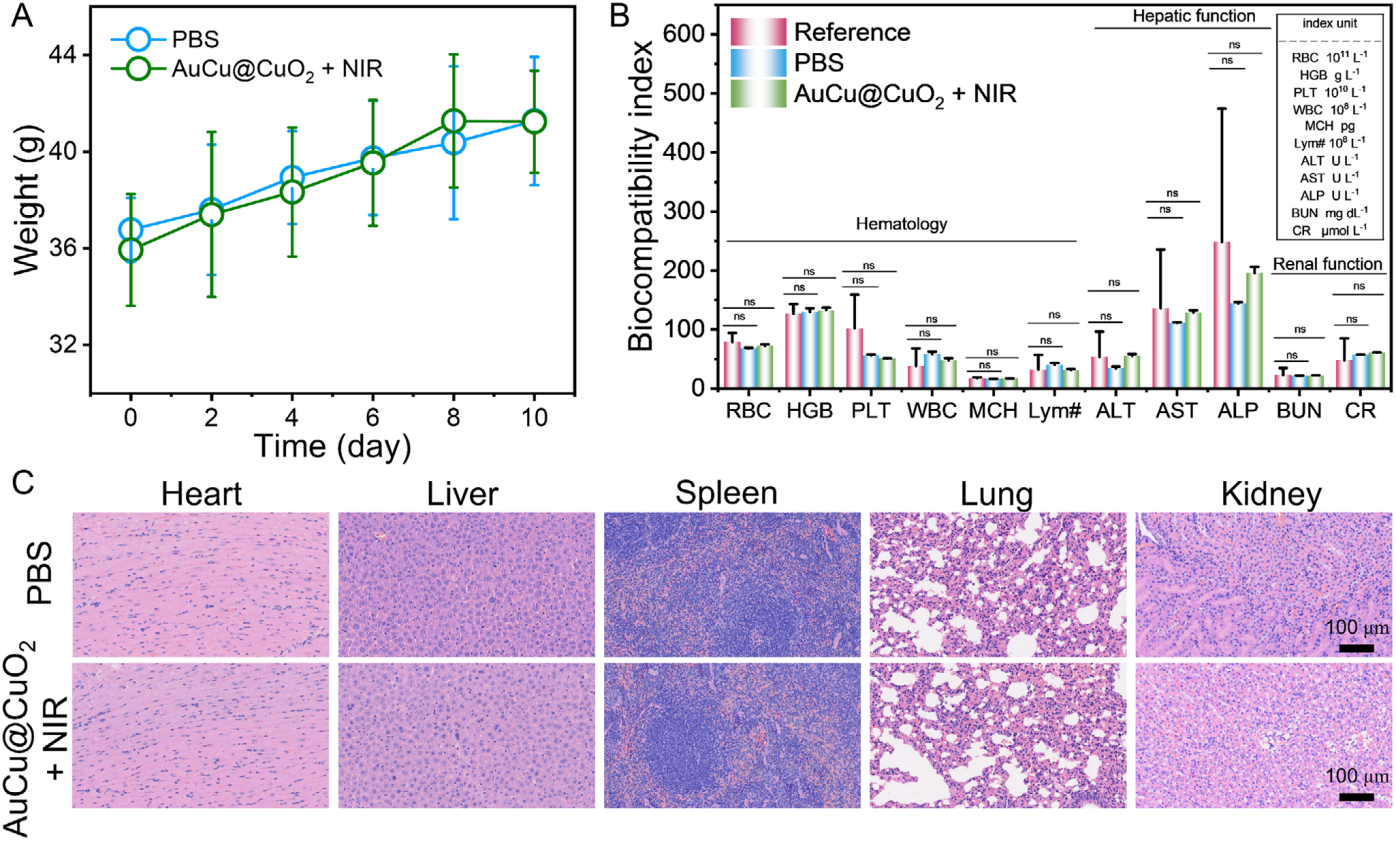
A) The body weight of mice, B) routine blood and biochemical indicators, and C) H&E staining of major organs in the PBS and AuCu@CuO_2_ +NIR groups. (The error bar represents the standard deviation from the repeated experiments after three times. *P*‐value: *P < 0.05, **P < 0.01, ***P < 0.001).

Finally, to evaluate the biocompatibility of AuCu@CuO_2_ aerogels, the body weight changes of mice in the PBS and AuCu@CuO_2_ aerogels + NIR groups were monitored. In the comparison of body weight, no substantial variation was observed between the AuCu@CuO_2_ aerogels + NIR and PBS groups (**Figure** **6**A). Blood specimens were gathered from the mice for hematological and biochemical analyses. The results indicated that all parameters were within normal ranges, with no significant differences between the AuCu@CuO_2_ aerogels + NIR and PBS groups (Figure 6B). Additionally, H&E staining of major organs (heart, liver, spleen, lungs, and kidneys) did not exhibit any evident pathological damage or inflammation in either the PBS or AuCu@CuO_2_ aerogels + NIR groups (Figure 6C). These results demonstrate a favorable biosafety profile of AuCu@CuO_2_ aerogels, indicating that they can be utilized safely and effectively as therapeutic agents for future applications.

## 3 Conclusions

In this work, we successfully developed an AuCu@CuO_2_ core–shell aerogel exhibiting multienzyme‐like activity and the ability to self‐supply H_2_O_2_ for the treatment of MRSA‐infected diabetic wounds. The AuCu@CuO_2_ aerogels demonstrate satisfactory photothermal properties, with an impressive photothermal conversion efficiency of 63.9% and outstanding photostability. Moreover, the self‐cascading mechanism, facilitated by the glucose oxidase‐like and peroxidase‐like activities of the AuCu@CuO_2_ aerogels, enables exceptional antibacterial efficacy by reducing blood glucose levels and generating hydroxyl radicals through an adequate in situ supply of H_2_O_2_. Furthermore, the catalase‐like activity of AuCu@CuO_2_ aerogels helps mitigate hypoxia by decomposing H_2_O_2_, thereby reducing oxidative stress and inflammation, promoting angiogenesis, collagen deposition, and neovascularization, and ultimately improving healing outcomes. This work highlights the significant therapeutic potential of strategies aimed at modulating the wound microenvironment for the treatment of infected diabetic wounds, providing valuable insights for wound management and paving the way for future research directions.

## 4 Experimental Section

The experimental section is available in the Supporting Information. All animal experiments were approved by the University of South China Animal Experiment Ethics Review and the Health Guide for the Care and Use of Laboratory Animals of National Institutes. The assigned accreditation number of the laboratory is 2023027.

### Statistical Analysis

The data were subjected to pre‐processing and statistically analyzed using OriginPro 2022 and ImageJ software. All data were obtained from at least three independent experiments and expressed as mean ± SD values. Error bars represent the standard deviation of three or four replicates. Sample size (*n*) for each statistical analysis was depicted in the figure legends. Analysis for two groups was calculated using an unpaired two‐tailed Student's t‐test; comparisons of more than two groups were determined via ANOVA‐LSD post hoc test. Symbols used include: “*” for *p* < 0.05, “**” for *p* < 0.01, and “***” for *p* < 0.001.

## Conflict of Interest

The authors declare no conflict of interest.

## Supporting information



Supporting Information

## Data Availability

The data that support the findings of this study are available from the corresponding author upon reasonable request.
