# Supplementary material for: AuCu@CuO2 Aerogels with H2O2/O2 Self‐Supplying and Quadruple Enzyme‐Like Activity for MRSA‐Infected Diabetic Wound Management
Source: Adv Sci (Weinh). 2025 Apr 27;12(27):2502391. doi: 10.1002/advs.202502391 (PMC12279186; doi:10.1002/advs.202502391)
Supplement: Supplementary file 1 — Supporting Information [file ADVS-12-2502391-s001.docx]

Supporting Information

AuCu@CuO_2_ Aerogels with H_2_O_2_/O_2_ Self-Supplying and Quadruple Enzyme-like Activity for *MRSA*-infected Diabetic Wound Management

*Xiaofeng Tan^1,2,#^, Nanyun Lin^2,#^, Sha Yang^2,3^, Hongyu Gong^2^, Minghui Wang^2^, Na Li^2^, Fen Liu^2^, Dajun Rao^2^, Yingying Wu^2^, Jing Tang^1,2^*, Qinglai Yang^1,2^**

^1^ Department of Anesthesiology, Hunan Provincial Maternal and Child Health Care Hospital & NHC Key Laboratory of Birth Defect Research and Prevention & MOE Key Lab of Rare Pediatric Disease, Hengyang Medical School, University of South China, Hengyang, Hunan, 421001, China.

^2^ Department of Hepatopancreatobiliary Surgery, The First Affiliated Hospital & Center for Molecular Imaging Probe, Cancer Research Institute, Hengyang Medical School, University of South China, Hengyang, Hunan 421001, China.

^3^ Pathology Research Group & Department of Pathology Institute of Basic Disease Sciences & School of Basic Medical Sciences, Xiangnan University, Chenzhou, Hunan 423000, China.

^#^ These authors contributed equally to this work.

Correspondence should be addressed to Dr. Jing Tang (233343848@qq.com) and Dr. Qinglai Yang (qingyu513@usc.edu.cn).

Keywords: Metallic aerogels; Multienzyme-like activity; *MRSA* infection; Diabetic wound healing; H_2_O_2_ and O_2_ self-supplying.

**Table of Contents**

[1. Materials and methods 3](#_Toc193732498)

[1.1 Chemicals 3](#_Toc193732499)

[1.2 Measurements 3](#_Toc193732500)

[1.3 Preparation of AuCu@CuO_2_ aerogels 3](#_Toc193732501)

[1.4 The Cu^2+^ and H_2_O_2_ release assay of AuCu@CuO_2_ aerogels 4](#_Toc193732502)

[1.5 The Multienzyme-like Activity of AuCu@CuO_2_ aerogels 5](#_Toc193732503)

[1.6 Photothermal performances of AuCu@CuO_2_ aerogels 6](#_Toc193732504)

[1.7 Bacterial cultivation and in vitro antibacterial tests 7](#_Toc193732505)

[1.8 Detection of reactive oxygen species (ROS) 8](#_Toc193732506)

[1.9 Bacteria Live/dead staining 8](#_Toc193732507)

[1.10 Bacterial morphology observation 8](#_Toc193732508)

[1.11 Crystal violet assay 9](#_Toc193732509)

[1.12. Biofilm live/dead staining 9](#_Toc193732510)

[1.13 In vivo Antibacterial Activity and Diabetic Wound Healing 10](#_Toc193732511)

[1.14 Statistical analysis 11](#_Toc193732512)

[2. Supplementary Figures 13](#_Toc193732513)

[3. Supplementary tables 27](#_Toc193732514)

[References 28](#_Toc193732515)

**1. Experimental Section**

1.1 Chemicals

Chloroauric acid (HAuCl_4_), copper(II) chloride dihydrate, potassium permanganate (KMnO_4_), dopamine, 3,3′,5,5′-tetramethylbenzidine (TMB), and methylene blue (MB) were purchased from Shanghai Titan Scientific Co., Ltd. The copper determination kit was obtained from Zhejiang Luheng Environmental Technology Co., Ltd. The live/dead bacteria staining kit was provided by Shanghai BestBio Scientific Co., Ltd. The American Type Culture Collection (ATCC) supplied the *E. coli* (ATCC 33694) and *MRSA* (ATCC 43300) strains. The 2,7-dichlorodihydrofluorescein diacetate (DCFH-DA) and glutathione (GSH) were acquired from Sigma-Aldrich Co., Ltd. (Shanghai, China). 5,5'-dithio-bis-(2-nitrobenzoic acid) (DTNB) was obtained from Aladdin Co., Ltd. (Shanghai, China).

1.2 Measurements

Transmission electron microscopy (TEM) images, elemental analysis, and selected-area electron diffraction (SAED) patterns were captured using a JEOL JEM 2100F machine. X-ray photoelectron spectroscopy (XPS, Thermo K-Alpha) was conducted to examine surface electronic information. Dissolved oxygen content was detected using a JPBJ-608 portable dissolved oxygen meter. UV-vis spectra were recorded with a Mapada UV-3200S spectrometer. The Fotric 225s infrared thermal imaging camera recorded temperature fluctuations and thermal images under 1064 nm laser irradiation. Laser confocal images were obtained from a confocal laser scanning microscope (Zeiss LSM880 and LSM980). Bacterial morphology was observed using a Hitachi Regulus 8100 scanning electron microscope.

1.3 Preparation of AuCu@CuO_2_ aerogels

Firstly, AuCu aerogels were primarily prepared via the one-step reduction method. Briefly, HAuCl_4_ (400 μL, 1%) solution, CuCl_2_·2H_2_O (3.2 mg), and dopamine (10 mg) were added into 30 mL anhydrous ethanol. The mixture solution was steadily heated to 60 °C under stirring. Next, the freshly prepared NaBH_4_ (0.05 M, 3 mL) aqueous solution was added under stirring. Subsequently, the mixture was kept motionlessly for 1 h at 60 °C, with abundant black flocculus-like solid formed. After centrifugation, water washing, and freeze-drying, AuCu aerogels were obtained for further modification.

The synthesis of AuCu@CuO_2_ aerogels employed an in-situ growth method. Briefly, polyvinylpyrrolidone (0.3 g), CuCl_2_ (0.01 M, 5 mL), NaOH (0.02 M, 5 mL), H_2_O_2_ (30%, 100 μL), and the AuCu aerogels (8 mg) was sequentially added to water (10 mL). Following a 30 min agitation, the resultant mixtures were subjected to centrifugation, water washing, and freeze-drying to obtain AuCu@CuO_2_ aerogels.

1.4 The Cu^2+^ and H_2_O_2_ release assay of AuCu@CuO_2_ aerogels

The copper assay kit was utilized to determine the amount of Cu^2+^ released by AuCu@CuO_2_ aerogels. Briefly, a standard curve was determined by varying the concentration of Cu^2+^ (0.4~5 mg·L^-1^) by assessing the absorbance change by a UV-vis spectrophotometer. Afterwards, the absorbance of AuCu@CuO_2_ aerogels under acidic environments was determined, and the release amount of Cu^2+^ was quantified using the standard curve.

The H_2_O_2_ production characteristics of AuCu@CuO_2_ aerogels were examined by KMnO_4_ colorimetric assay. The AuCu@CuO_2_ aerogels (1 mg·mL^-1^, 100 μL) were reacted for 10 min with the KMnO_4_ solution (50 μg·mL^-1^, 1 mL) and H_2_SO_4_ (0.1 M). The absorbance at 400 to 650 nm was measured using a spectrophotometer.

1.5 The multienzyme-like activity of AuCu@CuO_2_ aerogels

The 3,3’,5,5’-tetramethylbenzidine (TMB) was used as a classic colorimetric substrate to assess the peroxidase-like (POD-like) activity of AuCu@CuO_2_ aerogels. TMB (DMSO, 10 mM, 150 μL), AuCu@CuO_2_ aerogels (1 mg·mL^-1^, 150 μL), and HAc-NaAc buffer (pH 4, 700 μL) were mixed for 3 minutes at the optimal temperature. The absorption spectra were then monitored using a UV-vis spectrophotometer. The effects of varying experimental conditions, such as nanozyme concentration, reaction pH, and temperature, on the POD-like activity of AuCu@CuO_2_ aerogels were studied. Additionally, the generation of hydroxyl radicals (•OH) by AuCu@CuO_2_ aerogels was evaluated using the methylene blue (MB) assay. After mixing AuCu@CuO_2_ aerogels (5 mg·mL^-1^, 500 μL), MB (0.1 mM, 200 μL), and HAc-NaAc buffer (2.3 mL), the spectra were monitored at different reaction times using a UV-vis spectrophotometer.

The catalase-like (CAT-like) activity of AuCu and AuCu@CuO_2_ aerogels was evaluated by determining the dissolved O_2_ produced from H_2_O_2_ in the presence of metallic aerogels. The produced O_2_ was quantified using a portable dissolved oxygen meter. Briefly, AuCu@CuO_2_ aerogels (15 mg/mL, 200 μL) were added to 50 mL of H_2_O_2_ at various concentrations (0, 20, 50, 100, 200, and 500 mM), and the O_2_ produced was monitored by inserting a detector stick into the mixture.

The 5,5’-dithio-bis(2-nitrobenzoic acid) (DTNB) probe was used to test the glutathione peroxidase-like (GPX-like) activity of AuCu@CuO_2_ aerogels. Briefly, AuCu@CuO_2_ aerogels at different concentrations (100, 200, and 400 μg·mL^-1^, 4.5 mL) were treated with GSH (10 mM, 0.5 mL). Then, 100 μL of the reaction solution at different times was mixed with PBS (pH 7.4, 900 μL). Finally, a DTNB (10 mM, 5 μL) solution was added to the mixed solution and reacted for 3 minutes. The absorption peak at 412 nm was monitored to assess the GPX-like activity of AuCu@CuO_2_ aerogels.

The glucose oxidase-like (GOx-like) activity of AuCu@CuO_2_ aerogels was verified by detecting the H_2_O_2_ produced in the GOx oxidation reaction. Briefly, glucose solutions (100 mM, 800 μL) were mixed with AuCu@CuO_2_ aerogels (5 mg·mL^-1^, 200 μL) at a pH of 7.4. The reaction was incubated in a shaker at 37 °C overnight. The culture solution was centrifuged to obtain the supernatant. Then, 600 μL of the supernatant, HRP (200 μg·mL^-1^, 200 μL), and TMB (10 mM, 200 μL) were mixed and reacted for 3 minutes.

1.6 Photothermal performances of AuCu@CuO_2_ aerogels

The photothermal performances of AuCu@CuO_2_ aerogels were investigated under 1064 nm laser irradiation. Firstly, AuCu@CuO_2_ aerogels with different concentrations (25-200 μg·mL^-1^) were exposed to the laser with a power intensity of 1 W·cm^-2^. The infrared thermography garnered the temperature changes and thermal images. The influence of various laser power densities (0.5-1.5 W·cm^-2^) on the photothermal properties of AuCu@CuO_2_ aerogels was further explored. Additionally, the photothermal stability of AuCu@CuO_2_ aerogels was assessed through five laser on/off switch cycles. The photothermal conversion efficiency (PCE, η) was determined using the following formula^[1]^:

$$\eta=\frac{hs\left( T_{max}-T_{surr} \right)-Q_{dis}}{I(1-{10}^{-A_{1064}})}$$

Where *h* represents the thermal transmittance, *S* is the surface area of the sample container, *T_max_* is the maximum stabilization temperature reached by the aerogels, *T_surr_* is the surrounding room temperature, *Q_dis_* symbols the thermal energy generated by light absorption, *I* is the laser power, and *A_1064_* corresponds to the absorbance of AuCu@CuO_2_ aerogels at 1064 nm.

1.7 Bacterial cultivation and in vitro antibacterial tests

In order to validate the antibacterial effect of AuCu@CuO_2_ aerogels against Gram (+) and Gram (-) bacteria *in vitro*, we chose *MRSA* and *E. coli* as their representative strains. Luria-Bertani (LB) medium was seeded with the strain of *MRSA* or *E. coli*. Afterwards, when the bacteria were shaken at 37 °C until reaching the logarithmic growth period (12-16 h, OD_600_≈0.5), they were randomly divided into 8 groups and subjected to different treatments: (Ⅰ) PBS, (Ⅱ) PBS + NIR, (Ⅲ) AuCu aerogels, (Ⅳ) AuCu aerogels + NIR, (Ⅴ) AuCu@CuO_2_ aerogels, (Ⅵ) AuCu@CuO_2_ aerogels + NIR. Fresh bacterial suspensions were added to each group, incubating at 37 °C for 40 min, where the final concentrations of antibacterial agents and bacteria were 100 μg·mL^-1^ and 1×10^7^ CFU·mL^-1^. Finally, the bacterial suspension was diluted 30,000 times with sterile PBS and then evenly spread onto agar plates. The plates were then placed in a bacterial incubator for 16 h. After incubation, the colony-forming units (CFUs) on each agar plate were counted. Bacterial survival rate (%) = the colony number of the experimental group / the colony number of the control group × 100%.

1.8 Detection of reactive oxygen species (ROS)

The levels of ROS in each group were investigated through the fluorescence probe 2',7'-dichlorodihydrofluorescein diacetate (DCFH-DA). DCFH-DA probe (3 μL, 20 μM) was added to the bacterial suspensions of various groups dyeing 30 min. Then, the suspensions were subjected to laser irradiation (1064 nm, 1 W·cm^-2^) for 10 min or not. Afterward, the suspensions were washed three times using sterile PBS to remove excess dye and re-suspended in sterile PBS for imaging (*λ_ex_* = 488 nm, *λ_em_* = 525 nm).

1.9 Bacteria Live/dead staining

A live/dead staining kit, which includes N01 for labeling live bacteria and PI for labeling dead bacteria, was employed to assess bacterial viability using a confocal microscope. Briefly, E. coli and MRSA were treated with different treatments and washed three times with sterile 0.85% NaCl. Then, N01 (1 μL) and PI (2.5 μL) were added to stain the bacteria (30 minutes). Subsequently, the unbound dye was removed by rinsing the bacterial suspension three times with sterile 0.85% NaCl. The stained images of live/dead bacteria (green/red fluorescence) were recorded using confocal laser scanning microscopy. N01 excitation wavelength: *λ_ex_* = 488 nm, emission wavelength: *λ_em_* = 530 nm; PI excitation wavelength: *λ_ex_* = 535 nm, emission wavelength: *λ_em_* = 630 nm.

1.10 Bacterial morphology observation

After different treatments, bacterial morphology images were obtained using scanning electron microscopy (SEM). Bacterial suspensions from the different groups of E. coli and MRSA were centrifuged (3000 rpm, 5 minutes) to remove the supernatant. The collected precipitate was soaked in 2.5% glutaraldehyde (4 °C, 12 hours). The suspensions were then dehydrated sequentially in ethanol (10, 30, 50, 70, 90, 100%) for 10 minutes each. Finally, the bacteria were re-suspended in anhydrous ethanol, observed, and recorded using SEM.

1.11 Crystal violet assay

Freshly cultured bacterial suspensions (100 μL) were added to 96-well plates and incubated for 48 hours to form bacterial biofilms. The biofilms were then treated and placed in a bacterial incubator for 4 hours. Afterward, the biofilms were treated with paraformaldehyde (30 minutes), stained with crystal violet (30 minutes), and rinsed with PBS to visualize the bacterial biofilms. Finally, the biofilm was decolorized with 95% ethanol for 15 minutes and quantified using a spectrophotometer. The residual rate of bacterial biofilm (%) = OD_590_(the experimental group) / OD_590_ (the control group) × 100 %.

1.12. Biofilm live/dead staining

The bacterial suspension, grown to the logarithmic phase, was spread to cover the bottom of the confocal dish, and incubated in a bacterial incubator at 37 °C for 48 hours to allow biofilm formation. The cultured biofilm was subjected to various treatments, and each group was stained by adding 5 μL of N01 and PI dyes, respectively. The samples were incubated in the dark for 30 minutes to ensure complete staining of the bacteria. Finally, the biofilms were photographed in 3D mode using a laser confocal microscope, with all z-stacks collected at a thickness of 1.41 μm per layer.

1.13 In vivo Antibacterial Activity and Diabetic Wound Healing

All animal experiments in this study complied with the "Guidelines for the Care and Use of Laboratory Animals at the University of South China" and were approved by the Animal Ethics Committee of the University of South China. A diabetic wound healing model was established using male KM mice (8-10 weeks, 30-45 g) through chemical induction. Mice were fasted overnight and intraperitoneally injected with streptozotocin (STZ) at a dose of 150 mg·kg⁻¹. Mice were then allowed to feed normally, with daily monitoring of their body status and blood glucose levels. When the mice exhibited physiological symptoms such as polydipsia and polyuria, and their blood glucose level exceeded 16.7 mmol/L, the diabetic mouse model was deemed successfully established. Subsequently, the diabetic mice were subjected to isoflurane anesthesia, and an 8 mm circular wound was created on the right dorsal skin. A 50 μL suspension of MRSA (1×10⁷ CFU·mL⁻¹) was then applied to the wound to induce infection. One day later, the successfully infected mice were randomly assigned to different treatment groups: (i) PBS, (ii) PBS + NIR, (iii) AuCu@CuO_2_ aerogels, (iv) AuCu@CuO_2_ aerogels + NIR. For the AuCu@CuO_2_ aerogels + NIR group, 100 μL of AuCu@CuO_2_ aerogels and 900 μL of PBS were mixed to form a therapeutic agent (final concentration: 100 μg·mL⁻¹, final volume: 1 mL). Subsequently, the therapeutic agent (50 μL) was applied to the established wound and irradiated with a 1064 nm laser at 1 W·cm⁻² for 10 minutes (the unirradiated groups were treated in the dark for 10 minutes). Thermal images of the wounds were recorded with a thermal imaging camera every two minutes. After treatment, the wound diameter in each group was measured every two days. On the 15th day, the mice were euthanized to obtain fresh wound skin tissue. The tissue was first immersed in PBS, followed by ultrasound treatment to collect bacteria for plate counting experiments. The wound tissue was then fixed in formalin for 24 hours, and Hematoxylin-eosin (H&E) and Masson staining were performed to assess wound healing progress and study skin tissue regeneration and collagen deposition. Tissue samples were further stained immunohistochemically with CD86, CD163, TNF-α, IL-10, CD31, and α-SMA according to standard protocols. To evaluate the biosafety of AuCu@CuO_2_ aerogels, healthy KM mice were subjected to two different treatments: PBS and AuCu@CuO_2_ aerogels + NIR. The daily weight of the mice was recorded over a 14-day period. The mice were euthanized on the 15th day, and blood samples were collected for biochemical analysis. The vital organs (heart, liver, spleen, lungs, and kidneys) were extracted for H&E staining.

1.14 Statistical analysis

The data were processed and statistically analyzed using OriginPro 2022 and ImageJ software. All data were obtained from at least three independent experiments and expressed as mean ± SD values. Error bars represent the standard deviation of three or four replicates. The sample size (n) for each statistical analysis is indicated in the figure legends. Statistical analysis for two groups was performed using an unpaired two-tailed Student's t-test, while comparisons of more than two groups were determined via ANOVA with an LSD post hoc test. Statistical significance was denoted as follows: “*” for p < 0.05, “**” for p < 0.01, and “***” for p < 0.001.

**2. Supplementary Figures**


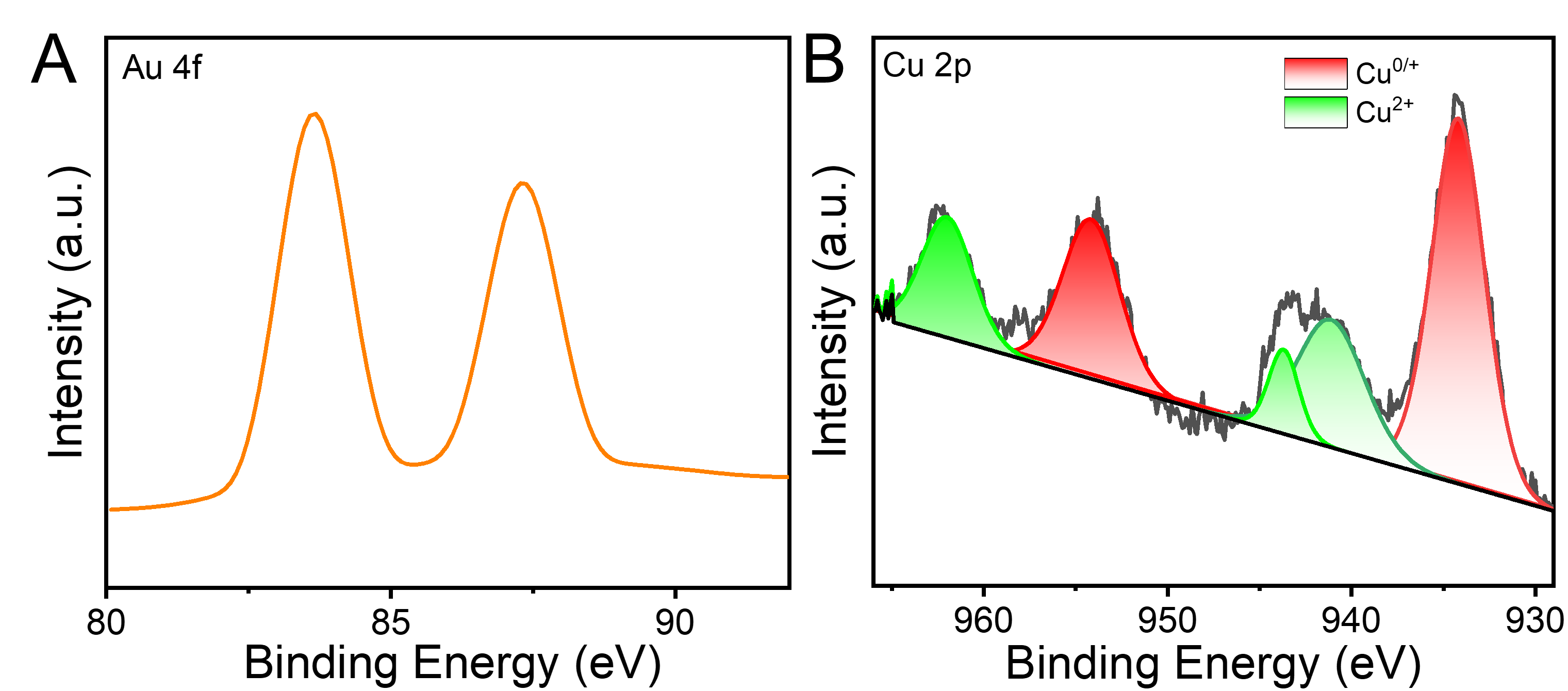


**Figure S1** High-resolution XPS spectra of Au 4f and Cu 2p for AuCu@CuO_2_ aerogels.


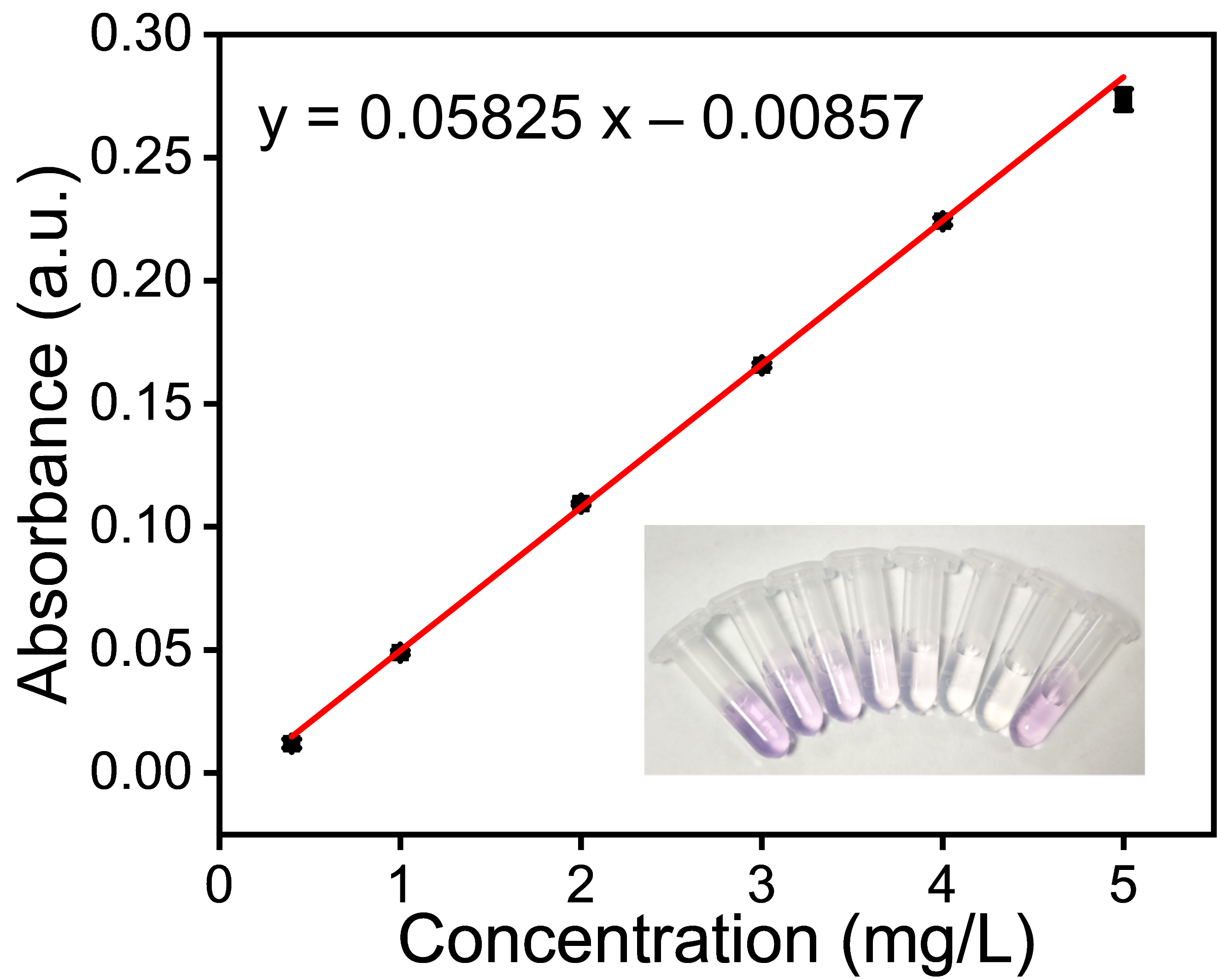


**Figure S2** Standard curves between Cu^2+^ concentration and absorbance using the detection kit. The inset picture from left to right shows the color change of Cu^2+^(0.4-5 mg·L^-1^), and the far right shows the color change of the release of Cu^2+^ for 1 mg·L^-1^ AuCu@CuO_2_ aerogels.


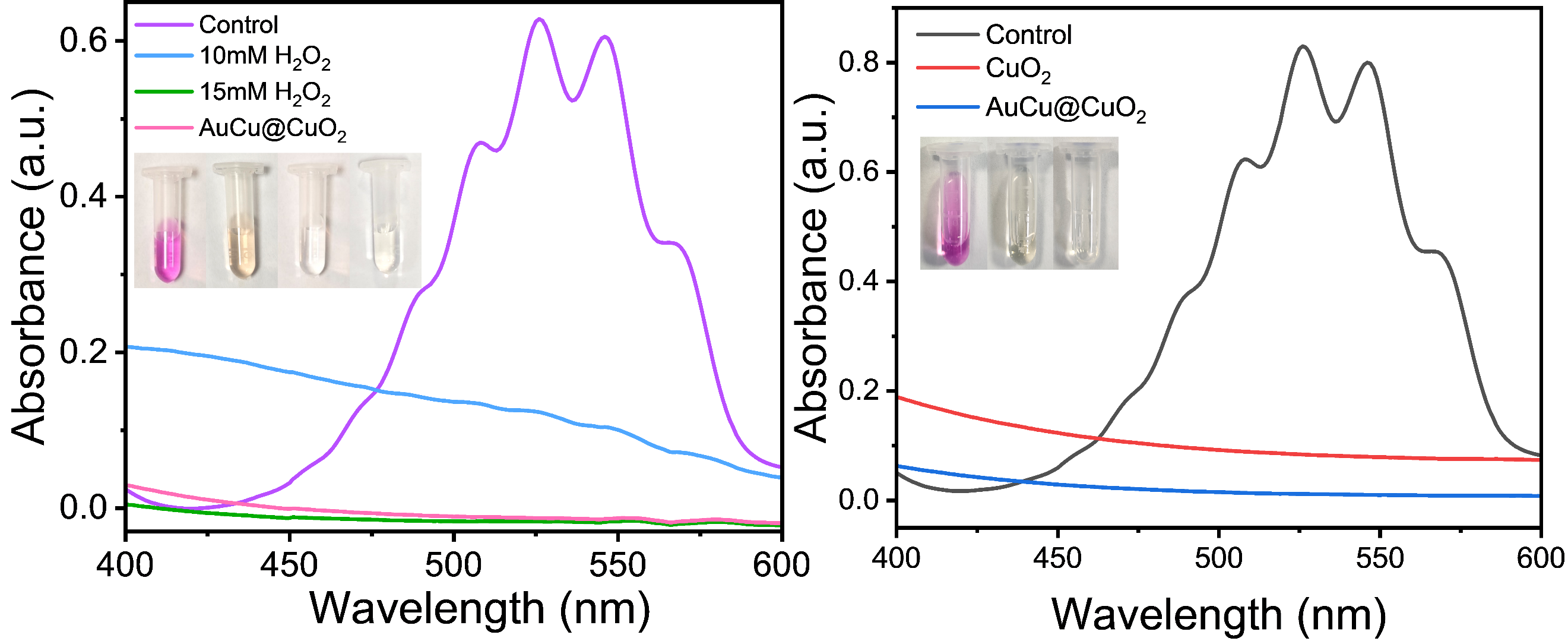


**Figure S3** UV-vis spectra of KMnO_4_ solution after different treatments.


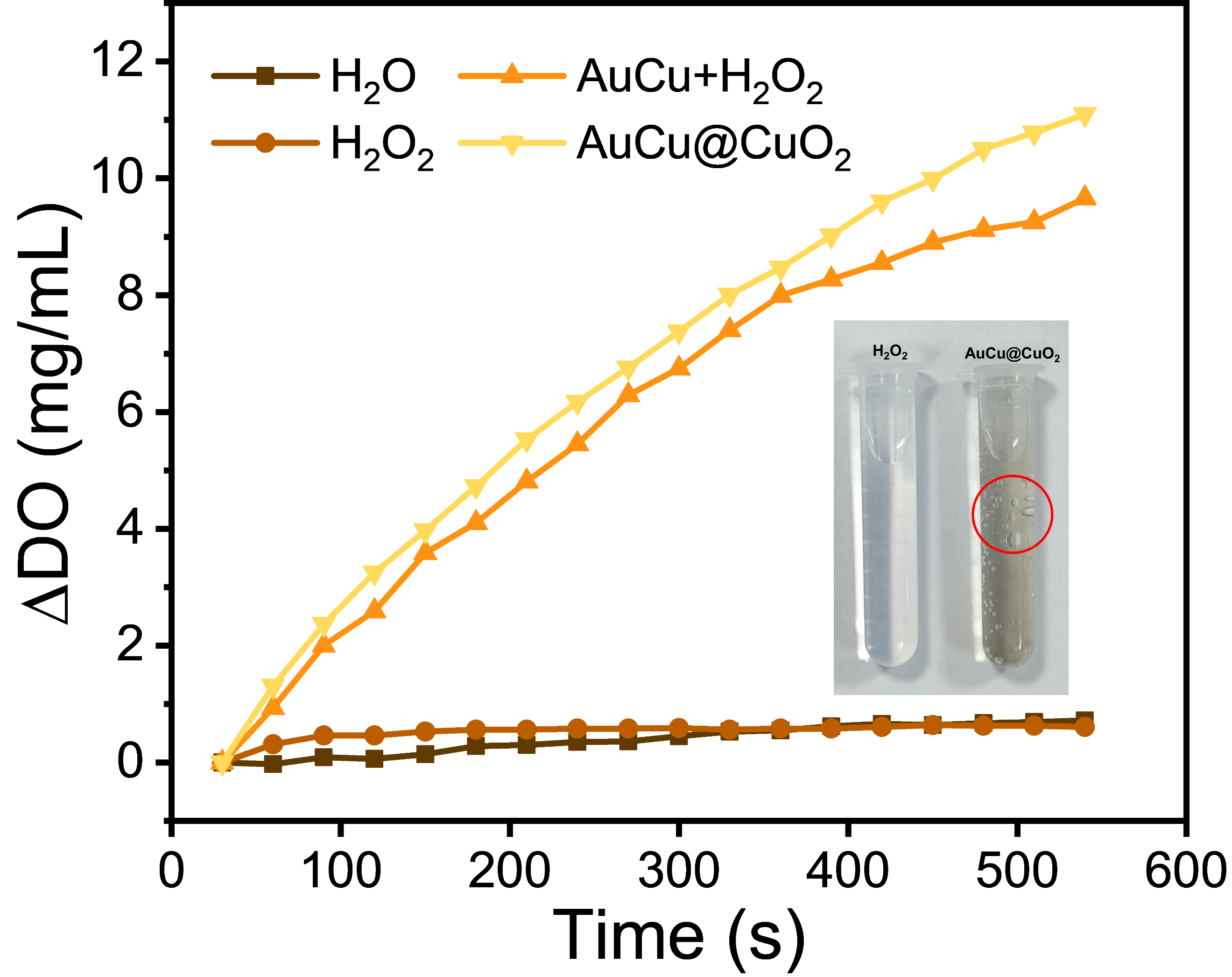


**Figure S4** the comparison of CAT-like activity between AuCu and AuCu@CuO_2_ aerogels. The inset indicated the formation of O_2_ bubbles by the decomposition of H_2_O_2_.


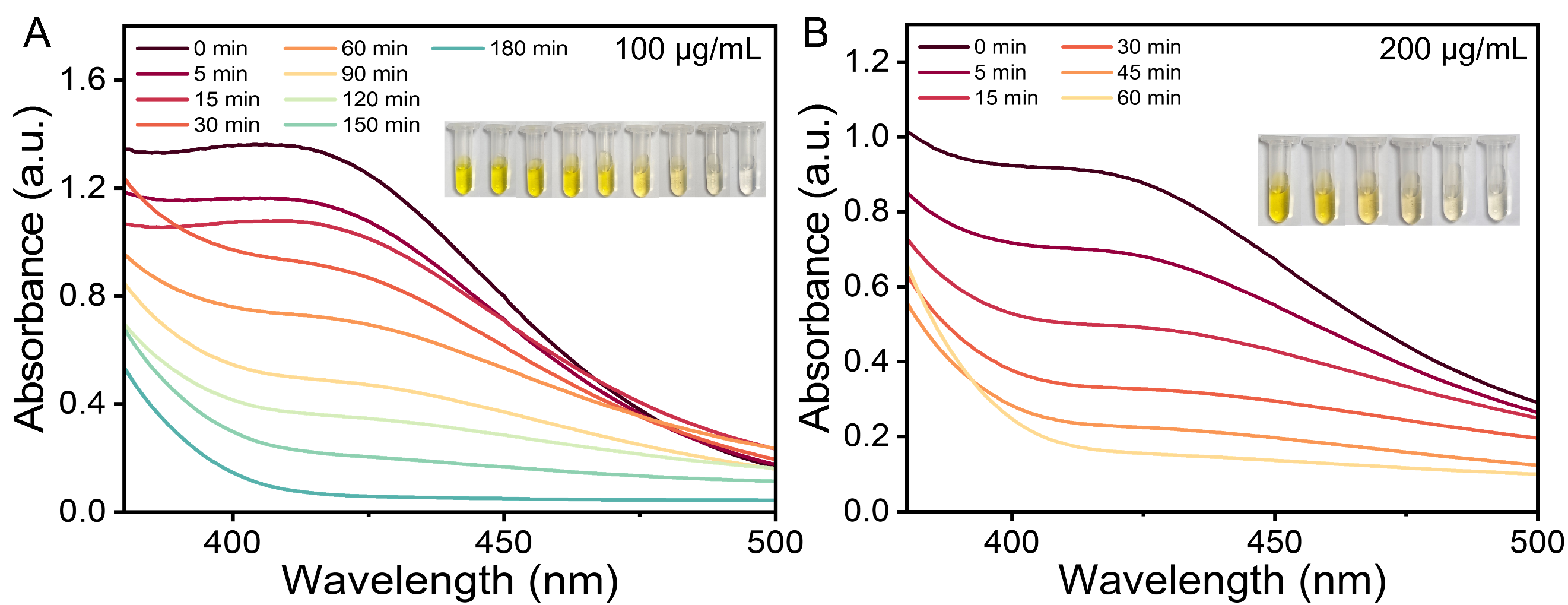


**Figure S5** The time-dependent GSH consumption in the existence of AuCu@CuO_2_ aerogels at a concentration of (A) 100 and (B) 200 μg·mL^-1^.


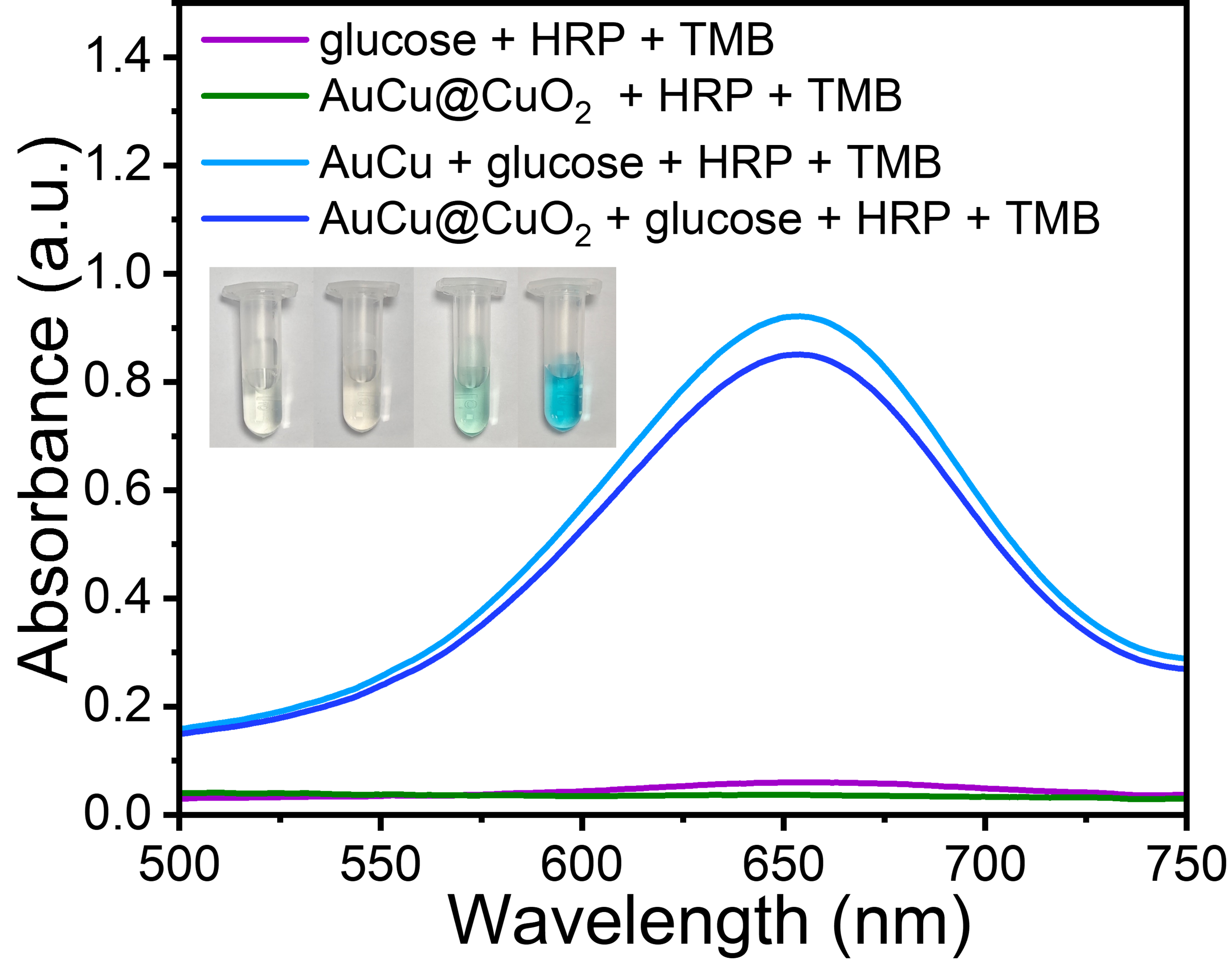


**Figure S6** Absorbance curves for the GOx-like activity of AuCu and AuCu@CuO_2_ aerogels. (The AuCu or AuCu@CuO_2_ aerogels were first incubated with glucose under neutral conditions, and the supernatants were purified and extracted to react with HRP and TMB under acidic conditions).


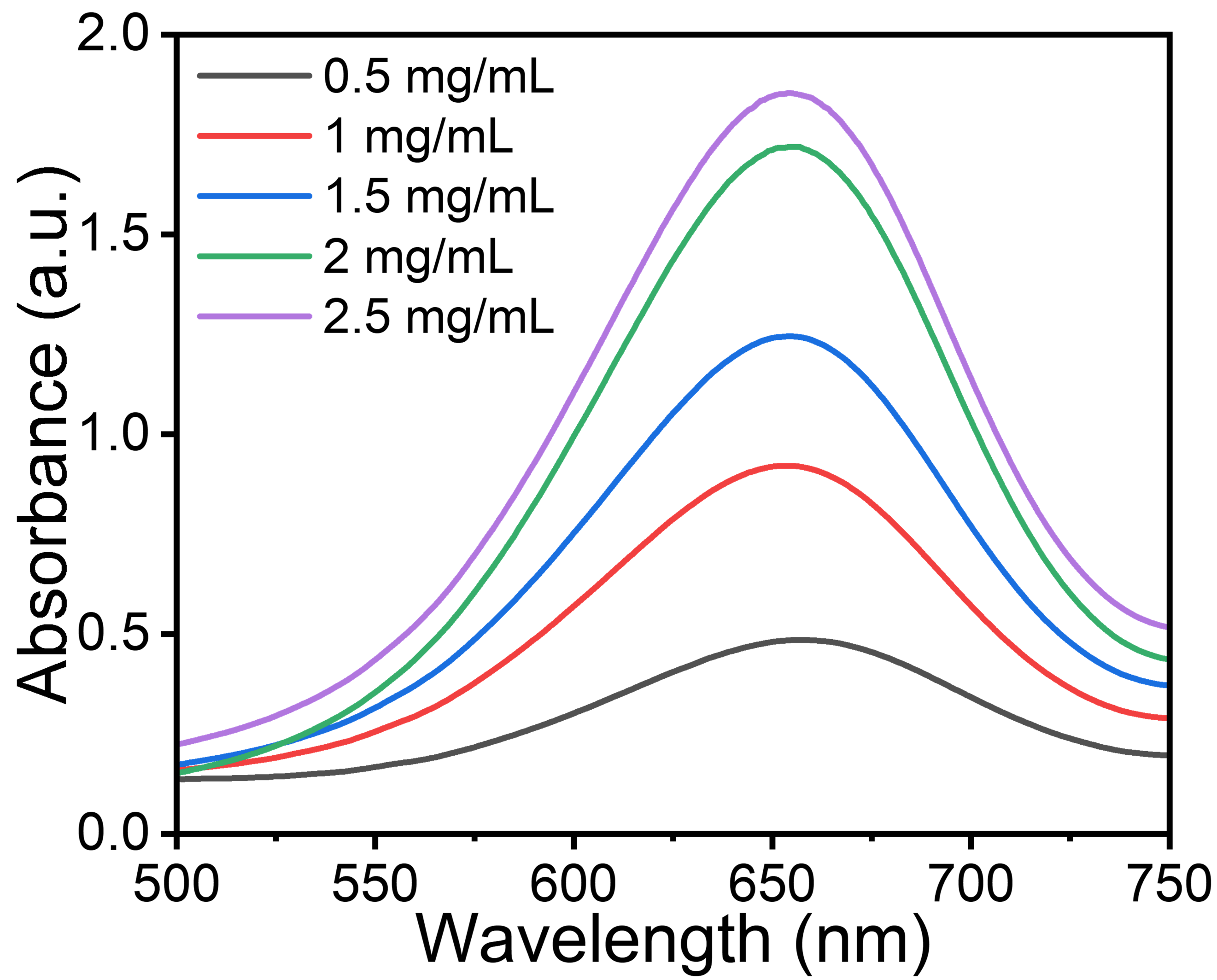


**Figure S7** Absorbance curves of the GOx-like activity under different concentrations of AuCu@CuO_2_ aerogels (0.5~2.5 mg·mL^-1^)


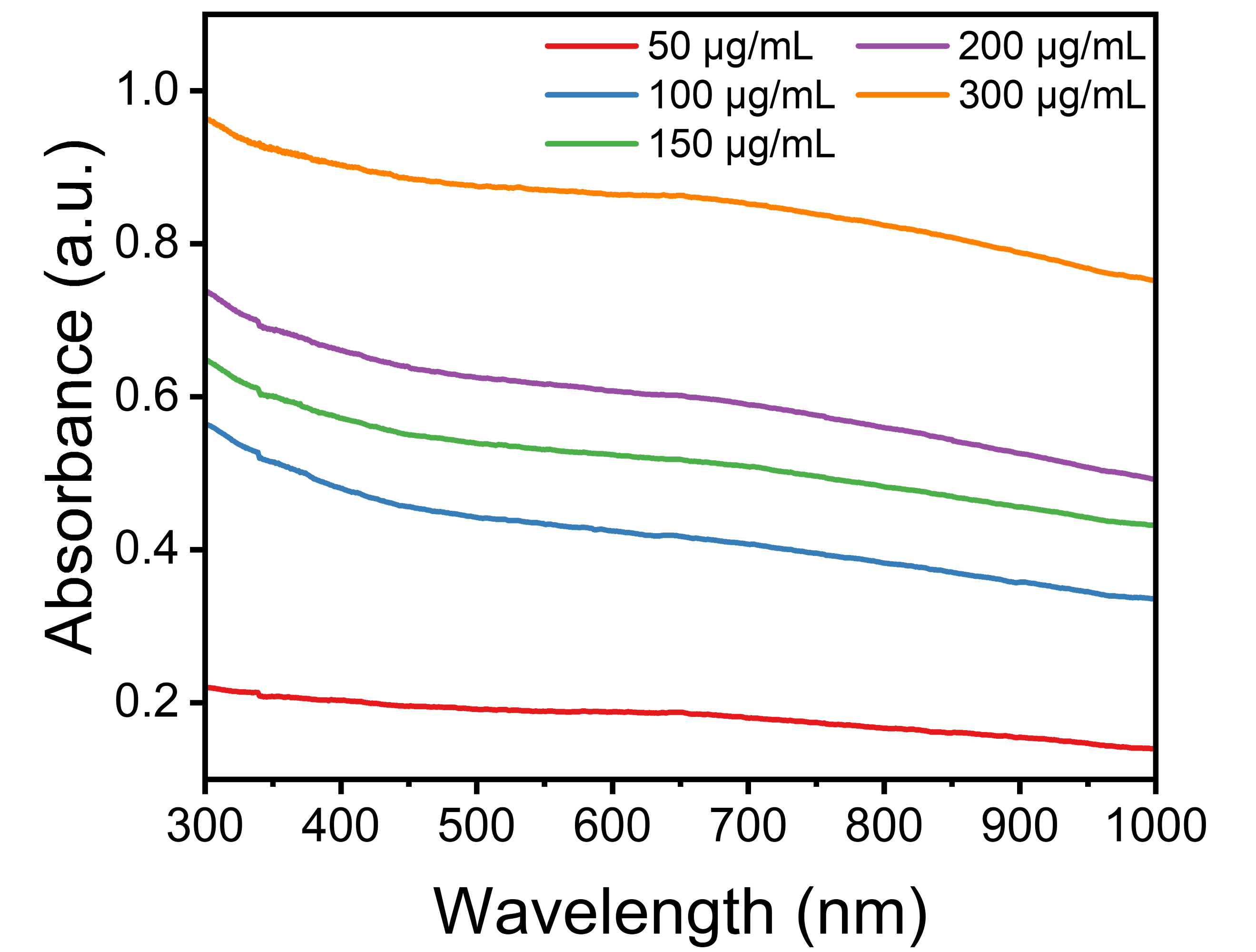


**Figure S8** UV-vis spectra of AuCu@CuO_2_ aerogels with different concentrations.


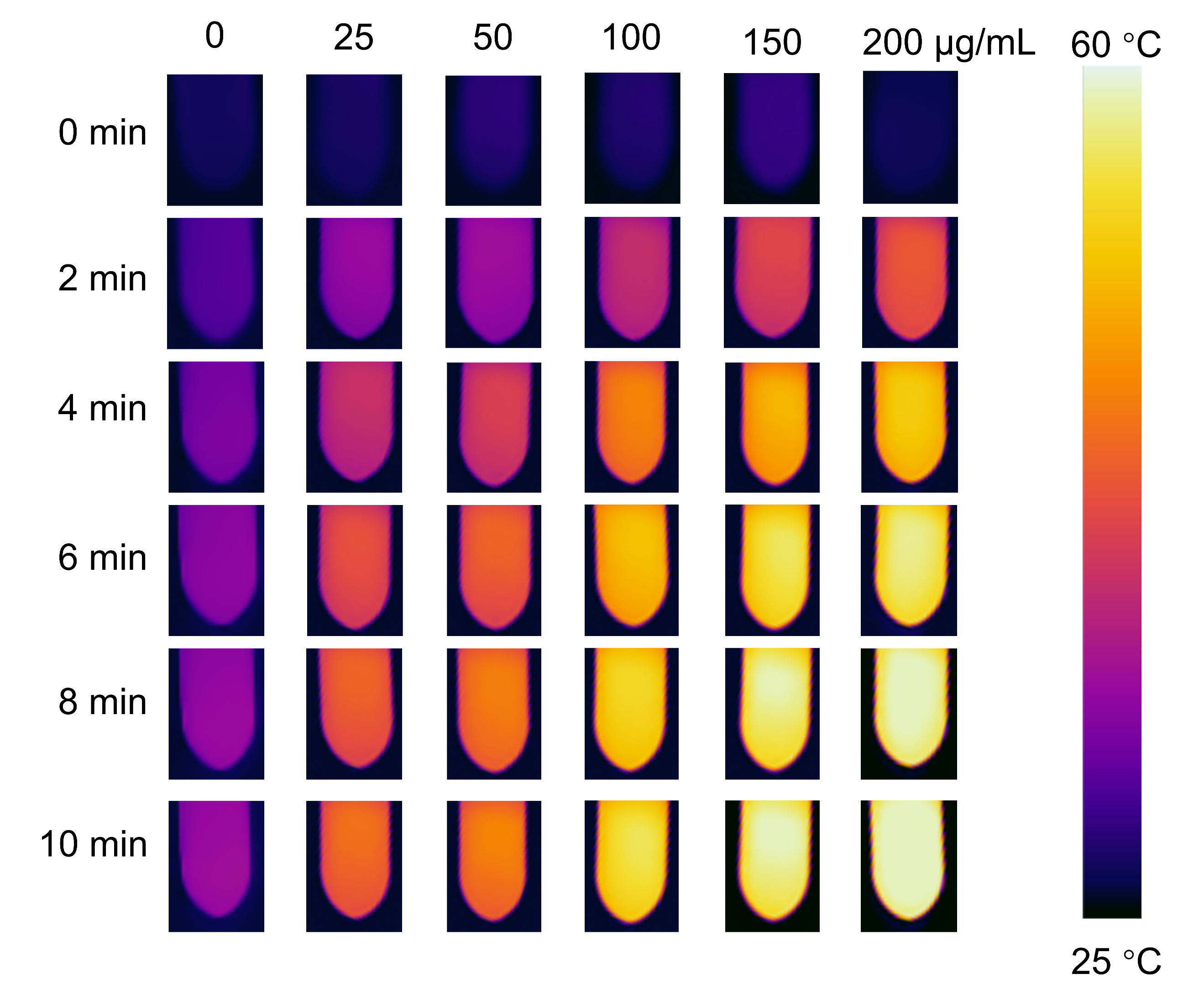


**Figure S9** The thermal images of AuCu@CuO_2_ aerogels with different concentrations under 1064 nm laser irradiation.


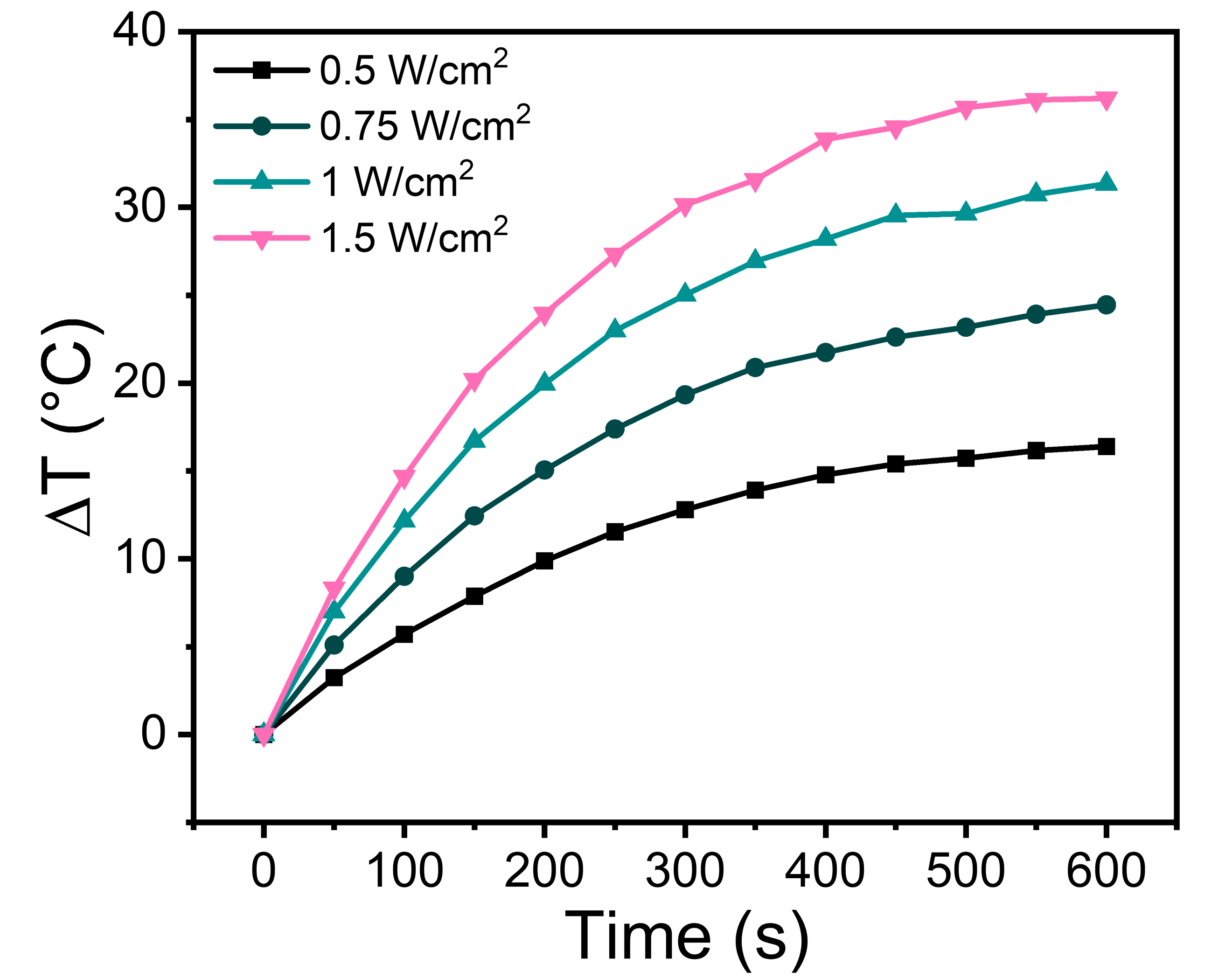


**Figure S10** The temperature-raising curves of AuCu@CuO_2_ aerogels at different laser power densities.


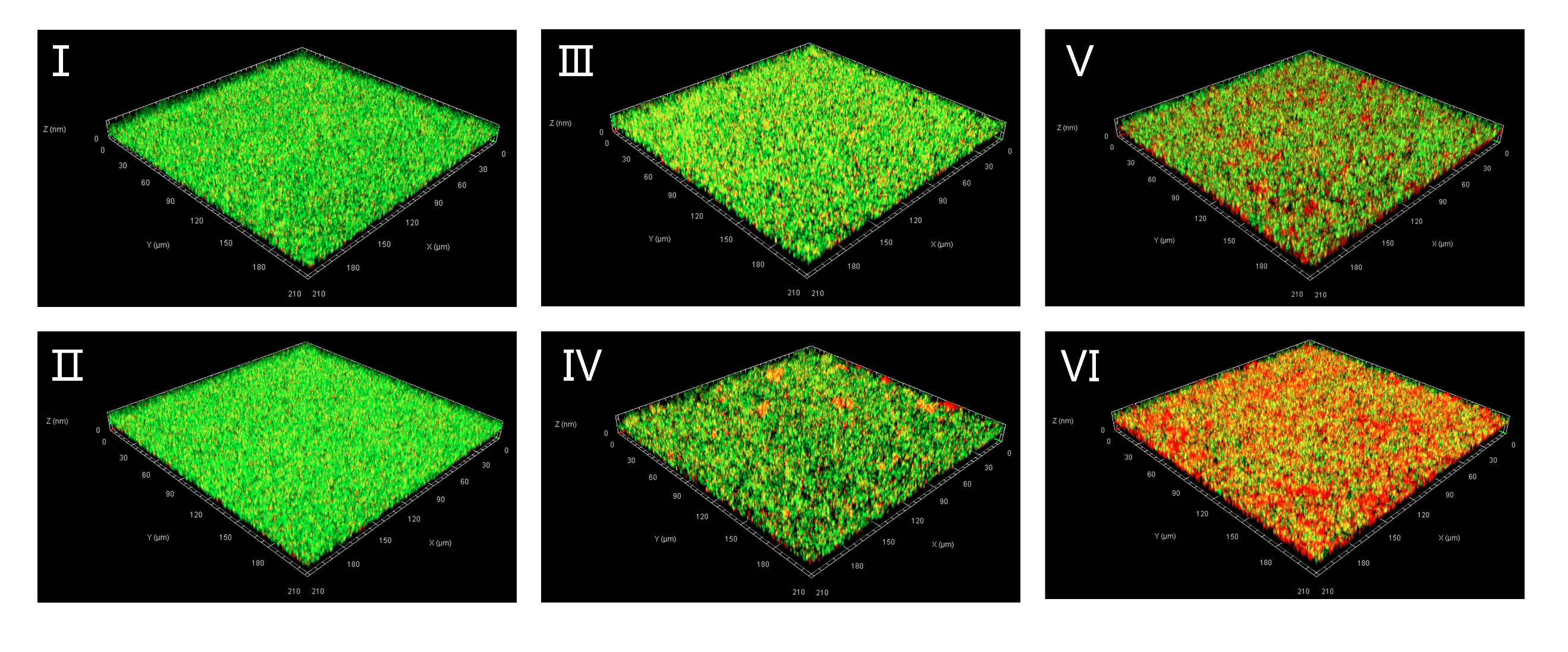
 **Figure S11** The 3D-CLSM images of the Live/Dead staining of *MRSA* biofilms in different groups.


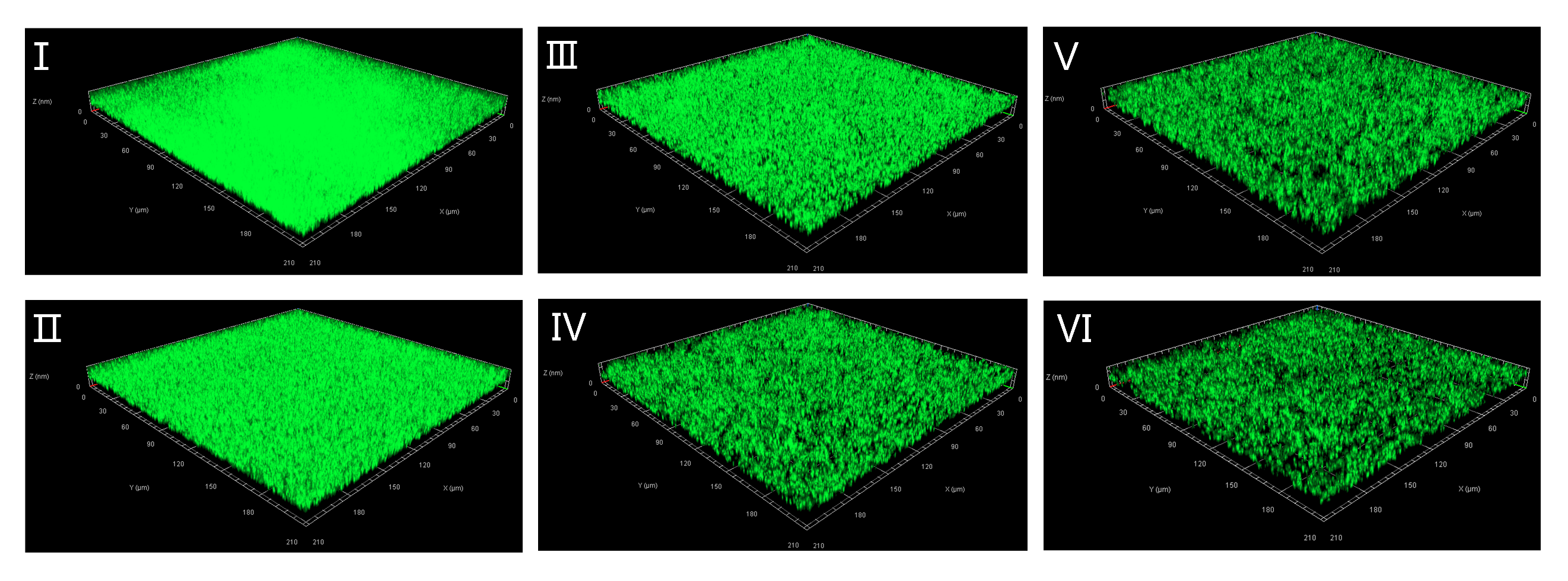


**Figure S12** The 3D-CLSM images of the changes in biofilm thickness in different groups.


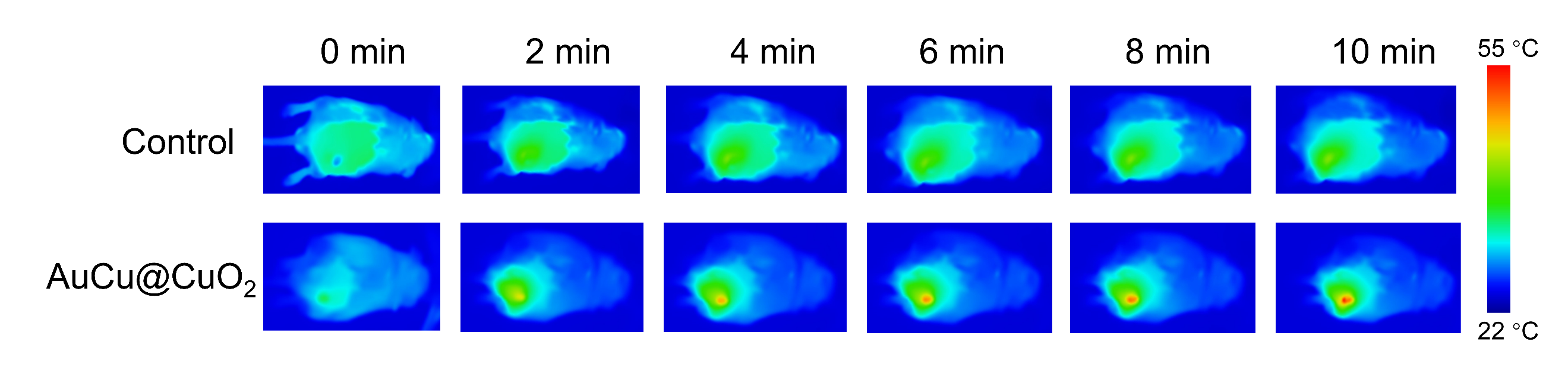
 **Figure S13** Photothermal images of *MRSA*-infected mice with different treatments under the 1064 nm laser irradiation.


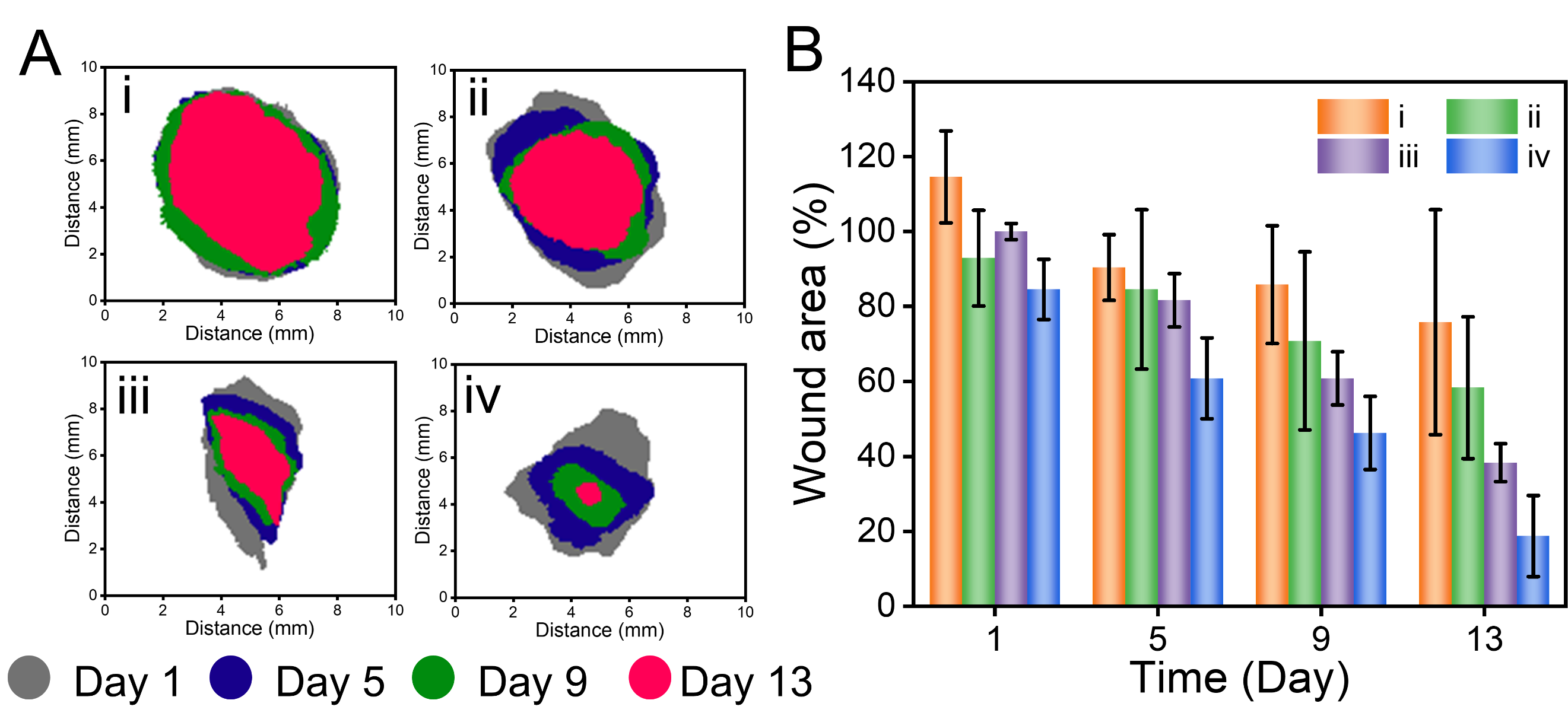


**Figure S14** (A) Traces of wound-bed closure during 13 days for different treatments and (B) corresponding changes in relative wound sizes.

# 3. Supplementary tables

**Table S1** Comparison of the photothermal conversion efficiency (η) between AuCu@CuO_2_ aerogels and other.

| Nanozymes | The photothermal conversion efficiency (%) | Reference |
| --- | --- | --- |
| AuCu@CuO_2_ aerogels | 63.9 | This work |
| Au−Cu JNSs | 42.14 | [2] |
| Cu_3_SnS_4_ | 55.7 | [3] |
| TA-Fe/Cu NPs | 33.4 | [4] |
| MXene@Fe_3_O_4_/Au/PDA | 48.7 | [5] |
| ATP@Au-CuNPs | 59.3 | [6] |

# References

[1] J. Li, D. Yang, W. Lyu, Y. Yuan, X. Han, W. Yue, J. Jiang, Y. Xiao, Z. Fang, X. Lu, *Adv. Mater.* **2024**, 36, 2405890.

[2] Q. Yang, H. Kong, L. Tang, Y. Ma, F. Liu, M. Liu, Y. Wang, P. Zhang, Y. Zheng, *ACS Appl. Nano Mater.* **2024**, 7, 20783-20792.

[3] Y. Yang, C. Wang, N. Wang, J. Li, Y. Zhu, J. Zai, J. Fu, Y. Hao, *J. Nanobiotechnology* **2022**, 20.

[4] X. Qin, R. Tian, B. Wang, H. Yang, J. Chen, X. Wang, J. Zhou, Q. Chen, J. Tian, Y. W. Yang, *Adv. Healthc. Mater* **2024**, 13.

[5] G. Liu, Q. Xiong, Y. Xu, Q. Fang, K. C.-F. Leung, M. Sang, S. Xuan, L. Hao, *Appl. Surf. Sci.* **2022**, 590.

[6] Q. Song, Y. Liu, P. Zhang, W. Feng, S. Shi, N. Zhou, X. Chu, J. Shen, *ACS Appl. Nano Mater.* **2022**, 5, 8621-8630.
